# Insertion torque recordings for the diagnosis of contact between orthodontic mini-implants and dental roots: a systematic review

**DOI:** 10.1186/s13643-016-0227-3

**Published:** 2016-03-31

**Authors:** Reint Meursinge Reynders, Luisa Ladu, Laura Ronchi, Nicola Di Girolamo, Jan de Lange, Nia Roberts, Annette Plüddemann

**Affiliations:** Department of Oral and Maxillofacial Surgery, Academic Medical Center, University of Amsterdam, Meibergdreef 9, 1105 AZ Amsterdam, The Netherlands; Via Matteo Bandello 15, 20123 Milan, Italy; Department of Veterinary Sciences, University of Bologna, Via Tolara di Sopra 50, 40064 Ozzano dell’Emilia, BO Italy; Department of Oral and Maxillofacial Surgery, Academic Medical Center and Academisch Centrum Tandheelkunde Amsterdam (ACTA), University of Amsterdam, Meibergdreef 9, 1105 AZ Amsterdam, The Netherlands; Bodleian Health Care Libraries, University of Oxford, Cairns Library Level 3, John Radcliffe Hospital, Oxford, OX3 9DU UK; Centre for Evidence-Based Medicine, Nuffield Department of Primary Care Health Sciences, University of Oxford, New Radcliffe House, 2nd floor, Jericho, Oxford, OX2 6NW UK

**Keywords:** Diagnostic test accuracy, Implant, Screw, Root contact, Root proximity, Insertion torque, Systematic review, Orthodontics, Contacting authors

## Abstract

**Background:**

Most orthodontic mini-implants (OMIs) are inserted between dental roots. The prevalence of contacting these structures is high. Such contacts can cause permanent root damage and implant instability. Increased torque levels during implant insertion (the index test) could be a more accurate and immediate measure for diagnosing implant-root contact (the target condition) than radiographs (the reference standard) and could ultimately lead to a reduction or elimination of X-ray exposure. To address this issue, we asked three questions: (1) whether OMIs with root contact had higher insertion torque values than those without, (2) what is the accuracy of the index test compared with the reference standard to diagnose the target condition and what are the adverse effects of the index test, and (3) whether intermediate torque values have clinical diagnostic utility.

**Methods:**

Methods were conducted according to our published protocol, which was based on the PRISMA-P 2015 statement. We applied broad spectrum eligibility criteria that included randomized and non-randomized studies on clinical, animal, and cadaver models. Not including such models would be unethical because it could slow down knowledge creation on the adverse effects of implant insertion. We conducted searches in more than 40 electronic databases including MEDLINE and 10 journals were hand-searched. Grey literature and reference lists were also searched. All research procedures were conducted independently by three reviewers. Authors of selected studies were contacted to obtain additional information. Outcomes on the three different research models were analysed separately. Systematic error was assessed with the Cochrane ‘Risk of bias tool’ for non-randomized studies.

**Results:**

One clinical, two animal, and two cadaver studies fulfilled the eligibility criteria of the first research question. All studies and subgroups demonstrated higher insertion torque values for OMIs with the target condition than those without. Mean differences (MD) between these effect estimates were statistically significant in one beagle model (MD, 4.64; 95 % CI, 3.50 to 5.79) and three subgroups of cadaver studies (MD, 2.70; 95 % CI, 1.42 to 3.98) (MD, 3.97; 95 % CI, 2.17 to 5.78) (MD, 0.93; 95 % CI, 0.67 to 1.20). Highest mean differences were identified in most self-drilling compared with pre-drilling groups. Clinical heterogeneity between studies was high, and many items were underreported. All studies except one cadaver study scored at least one domain as ‘serious risk’ of bias. No studies addressed the second research question. One cadaver study addressed the third question which showed the importance of recording torque levels during the entire implant insertion process. Responses of contacted authors were helpful, but often difficult to obtain. Implants fractured in one animal and in one cadaver model.

**Conclusions:**

All eligible studies scored higher insertion torque values for implants with root contact than those without, but none of these studies assessed the diagnostic accuracy of the index test. The inclusion of non-randomized and animal and cadaver models in this systematic review provided key findings that otherwise would have been wasted. Such studies are important in the context of the wide applicability of this test, the high prevalence of the target condition, and the underreporting of adverse effects of interventions. A protocol for a potential new diagnostic pathway was presented, and the importance of contacting authors was addressed. The applicability of the findings should be interpreted in the context of underreporting and the many limitations of the included studies.

**Electronic supplementary material:**

The online version of this article (doi:10.1186/s13643-016-0227-3) contains supplementary material, which is available to authorized users.

## Background

Contact between orthodontic mini-implants (OMIs) and dental roots during the insertion process of these devices is a common problem because inter-radicular spaces are narrow [1–7]. Such contacts have been associated with root damage and increased implant failure rates [8–10].

An accurate test to diagnose implant-root contact is therefore indicated. To address this issue, we assessed whether OMIs with root contact (the target condition) had different insertion torque values compared with those without. Using specific insertion torque values (the index test) as a diagnostic test of the target condition could be more accurate and have less adverse effects compared with radiographic images (the current reference standard). A protocol for this review was published previously [11].

### The target condition

Orthodontists need some form of anchorage to resist the reciprocal forces of orthodontic tooth movement. Intra- or extra-oral removable appliances or connecting groups of teeth within or between dental arches are generally used for this purpose. These treatment mechanics are effective, but they often (1) depend on patient cooperation, (2) cannot prevent some loss of anchorage, and (3) have a limited area of application [12]. Most of these limitations do not apply to OMIs, but the placement of these devices requires a surgical intervention.

**Fig. 1:**
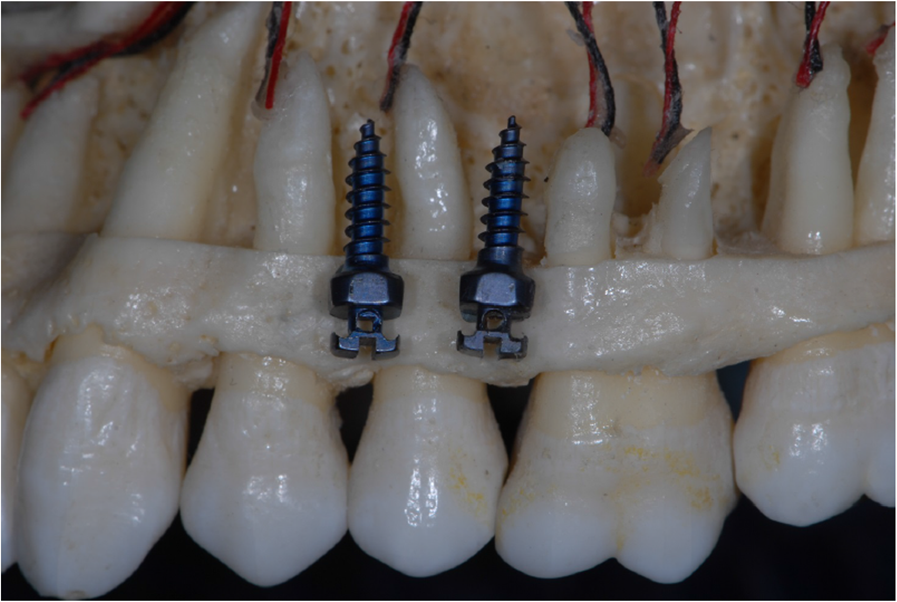
Inter-radicular distances in the maxillary arch and 1.5 mm (diameter) orthodontic mini-implants. Quattro implants PSM Medical Solutions; Tuttlingen, Germany

Implant-root contact is common during this insertion procedure because a minimum of 3 mm of inter-radicular distance has been recommended for safe implant insertion, and such dimensions are available in limited areas in both jaws (Fig. 1) [5, 7]. There is only a small margin for error because the most commonly used OMIs vary between 1.5 and 2 mm in diameter [9]. Various studies have recorded a high prevalence of implant-root contact. Kau et al. [13] observed contact between implants and the periodontal ligament in 65.2 % of consecutively inserted OMIs. Kim et al. [4] scored 30 % implant-root contact, and another clinical study recorded the target condition for 21.3 % of implants that were inserted by inexperienced operators and 13.5 % for experienced operators [2]. Motoyoshi et al. [6] recorded the target condition for 20.5 or 17.1 % for screws placed respectively with pre-drilling or self-drilling techniques. Almost identical findings were recorded by the same research group in other publications on this topic [3, 14]. Accurate positioning methods are available to reduce implant-root contact, but these techniques are complex, expensive, and require additional radiographs [4, 15–20].

Implant-root contact can cause extensive root damage [21]. This condition could further deteriorate because implants do not always remain stationary and can migrate towards the roots during orthodontic tooth movement [22–24]. The quality of healing of root damage after implant removal varies, and a damaged dental pulp is less likely to repair completely [8, 21, 25]. These issues have also been reported for inter-maxillary fixation screws used in maxillofacial surgery [26–28]. Various studies have also associated close implant-root contact with increased failure rates [4, 10, 29–31]. A recent systematic review recorded three times higher failure rates for OMIs with root contact compared with implants placed away from dental roots [9].

### The reference standard

**Fig. 2:**
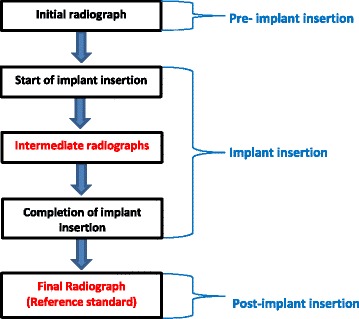
Current diagnostic pathway for assessing implant-root contact. The steps in red-type face can be eliminated when the index test is more accurate than the reference standard. This figure was based on a figure published in the protocol of this systematic review [11]

Clinicians generally take two or three radiographs during the current diagnostic pathway of implant insertion (Fig. 2). The first image is used to measure the available inter-radicular distance prior to implant insertion. Additional X-rays are taken during and at the completion of implant insertion to diagnose potential implant-root contact. The final radiograph is currently the reference standard to assess the target condition. Exposure to X-rays is the main disadvantage of this test. Operators therefore tend to avoid the intermediate radiograph and only take one radiograph, i.e. at the completion of the insertion procedure when damage to the root has already occurred.

### The index test

The index test of this systematic review does not have these shortcomings. This test measures torque values during the insertion of OMIs. Specific increased insertion torque values have been associated with implant-root contact [6, 25, 32, 33]. The American Society for Testing and Materials (ASTM International) defines insertion torque as follows: ‘the amount of torque required to overcome the frictional force between the screw and the material used for testing while driving the screw into the material’ [34].

The index test could be used as a ‘replacement’, a ‘triage’, or as an ‘add-on’ test [35]. The index test could replace the reference standard when it is more accurate, faster, cheaper, and causes less adverse effects, e.g. reduction or elimination of X-rays. The index test could be a candidate as a triage test to reduce the use of radiographs, e.g. in young patients that have undergone radiotherapy for cancer treatment. It could also be indicated as an ‘add-on’ test to improve diagnostic accuracy. The index test could also be crucial in the decision-making process because continuous recording of the implant insertion process could reveal the specific time-point of implant-root contact, e.g. a sudden increase in insertion torque values [32]. The clinician can then, for example, stop the insertion procedure, change the insertion path prior to complete implant insertion, and thereby prevent additional biologic damage and patient discomfort.

The index test does not (1) prolong the intervention, (2) require additional learning, or (3) introduce unexpected adverse effects because torque values are recorded during the standard insertion process with screwdrivers with built in torque sensors. Economic issues are also not expected because of the relatively low purchasing price and the shared use of the index test, i.e. insertion torque values are also measured to record primary stability of OMIs [10, 36].

Various issues on the importance of conducting the index test were addressed in the previous paragraphs. One can further reason that conducting this index test is particularly important in the context of the high prevalence of the target disorder and because most (90 %) OMIs are placed between dental roots [4, 6, 37]. Outcomes on the diagnostic accuracy of the index test can also have external validity to other specialties, e.g. maxillofacial surgery. In addition, no systematic reviews have addressed our research questions.

### Objectives

Three research questions were formulated to address the objectives of this systematic review [38, 39].

- Research question 1

‘Do OMIs with root contact have higher insertion torque values than those without this target condition?’

- Research question 2

‘In OMIs (participants or problem), what is the accuracy of the level of insertion torque values (index test) compared to radiography (reference standard) to distinguish those with and without implant-root contact (target condition)’. We also assessed the adverse effects of using this test.

- Research question 3

‘Do intermediate recordings of insertion torque values have clinical utility for the diagnosis of the target condition’?

## Methods

Prior to conducting this review, we developed and published a protocol of our research methods [11]. This protocol was designed according to the guidelines for conducting systematic reviews of diagnostic accuracy of the Diagnostic Test Accuracy Working Group of the Cochrane Collaboration, the Cochrane Handbook for systematic reviews of interventions, and the preferred reporting items described in the PRISMA-P 2015 statement [40–43]. The methods, results, and discussion of this systematic review comply with the PRISMA 2009 Checklist and follow its order (Additional file 1) [44, 45].

### Differences between protocol and review

We applied three key modifications to our protocol as were suggested by one of the peer reviewers of this systematic review.

1. we changed the order of the first two research questions to improve the flow of the article. This change needs to be considered when consulting the published protocol [11]. The phrasing and the content of the original questions was not revised.
2. we applied the Cochrane ‘Risk of bias tool’ for non-randomized studies [46]. The rationale for using this instrument was explained in the section ‘Risk of bias in individual studies’.
3. we only reported on methods that were ‘actually’ done. When methods were different from those planned in our protocol, we gave a brief justification.

### Eligibility criteria

To avoid inappropriate exclusion of relevant articles, we aimed for broad-scope inclusion criteria that were sufficiently specific and still covered our research objectives [47]. We adapted these criteria to the particular character of our research questions because (1) for ethical reasons, we did not expect to find randomized controlled trials in which torque values were measured after deliberately inserting OMIs into dental roots and (2) non-randomized studies on humans in which torque values were recorded with or without root contact could be under-represented in the literature. For these reasons, we also included in vivo animal studies and cadaver models. The importance of including these experimental models is further addressed in the ‘Discussion’ section under the subheading ‘Strengths and weaknesses of the systematic review’.

A detailed description of the eligibility criteria was presented in our protocol [11]. In addition, we excluded primary studies that met the eligibility criteria, but did not report on the outcomes of interest of this systematic review. This review is not registered in the PROSPERO database because this database only includes studies on human participants [48].

### Information sources and search

Eligible studies were searched in the period from 1 January 1997, the year of the introduction of OMIs in orthodontics, until 19 June 2015 [49]. The following electronic databases were searched:

- General and subject-specific electronic databases were consulted from PubMed (MEDLINE), Google Scholar Beta, EMBASE (Ovid), Science Direct, and Cochrane Central Register of Controlled Trials (CENTRAL) [50–52].
- Additional studies were searched through the ‘Related Articles’ feature in PubMed.
- TRIP Database, NHS Evidence, and SUMSearch2 were also searched.
- The following citation indexes were searched: Science Citation Index, Scopus, and Web of Science [50, 51].
- The following national and regional databases were also searched: African Index Medicus, African Journals online (AJOL), Australasian Medical Index, Index Medicus for the Eastern Mediterranean Region, IndMED, KoreaMed, LILACS, Index Medicus for the South-East Asia Region (IMSEAR), and Western Pacific Region Index Medicus (WPRIM) [50, 51].

A librarian, (NR), specialized in computerized searches of health care publications assisted with the development of the search strategy. A detailed protocol for developing this search strategy has been presented previously in our published protocol [11]. The full electronic search strategy for both PubMed and Google Scholar Beta was also given in this protocol. The search strategy for PubMed: (torque OR insertion torque OR torquing OR torqueing OR torque sensor* OR torque device* OR torquing device* OR torqueing device* OR torque screwdriver* OR torque driver*) AND (root* OR root contact* OR root vicinity OR dental root* OR root damage OR tooth OR teeth OR tooth contact* OR tooth vicinity) AND (implant* OR mini implant* OR micro implant* OR microimplant* OR screw* OR mini screw* OR miniscrew* OR micro screw* OR microscrew* OR temporary anchorage device*).

According to our protocol, we also consulted other resources, i.e. the grey literature, reference lists, and hand-searched key journals, and contacted pertinent stakeholders on our topic of interest [11].

### Study selection

Three topic experts (RMR, LL, and LR) independently selected the studies. In the case of disagreement on the eligibility of an article, reviewers reread and discussed the pertinent paper and, if necessary, contacted its authors [45]. Study selection was summarized using a PRISMA flow diagram, and all excluded articles were presented in a table together with the rationale for their exclusion [44, 45]. A detailed description of our study selection procedures and our methods for contacting authors were described in our published protocol [11].

### Data collection process and data items

The Standards for the Reporting of Diagnostic accuracy studies (STARD) checklist was consulted for the development of data extraction forms [53, 54]. Data collection forms of previous systematic reviews on OMIs were also checked for pertinent items [55–57]. All data collection forms were tailored to our specific research questions and subsequently pilot tested on a series of articles. These pilot tests were used to further fine-tune these forms and to calibrate the three reviewers (RMR, LL, and LR). Our data collection forms with a full description of the extracted data items, and all data extraction procedures were given in our protocol [11].

### Outcomes and prioritization

#### Research question 1

The differences between maximum insertion torque values of implants with or without the target condition were calculated for the first research question. Outcome measures were recorded in the original format as defined by the authors of the selected studies. These measures were transformed to the effect estimate of this systematic review, i.e. Newton centimetre (Ncm), after the completion of all data extraction procedures [58]. Differences in the type of target condition, e.g. with or without root penetration, and different time points and insertion depths for measuring these outcomes were subdivided in subgroups and assessed separately.

#### Research question 2

For the second research question, we conducted scoping searches of the literature to establish a threshold for test positivity. We identified one clinical and one animal study during these initial searches [6, 25]. Insertion torque values of OMIs with root contact in self-drilling groups increased respectively 22.5 % in human participants and 113 % in adult beagles compared with implants without this target condition [6, 25]. The former population consisted of a group of 79 (24 males and 55 females) adolescents and young adults that received a total of 143 OMIs in the same standardized location in the maxillary arch [6]. All patients were treated in the same setting, i.e. a university dental hospital in Tokyo, Japan. Based on these findings, we defined a hypothetical maximum insertion torque increase of 25 % or more as a positive result of the index test and values inferior to this threshold as a negative outcome. Reference test positive referred to implant-root contact, which included both touching (glancing) of the root by the OMI as well as penetration of the root. We do not further refer to our ‘planned’ research question 2 in the ‘Methods’ section because none of the identified studies in this systematic review addressed this diagnostic accuracy question [11].

#### Research question 3

For the third research question, we assessed whether sudden steep increases in torque values were identified during the implant insertion process. Adverse effects of the interventional procedures were also recorded.

### Risk of bias in individual studies

We used the Cochrane ‘Risk of bias tools’ for non-randomized studies (ACROBAT-NRSI) because our searches did not identify any randomized studies that addressed research questions 1 and 3 [46, 59]. The QUADAS-2 tool that was developed in our protocol [11] was also not applied because this instrument was specifically developed to assess risk of bias for research question 2 and no eligible studies addressed this question [60–62].

### Summary measures

The mean insertion torque values with their standard deviation for OMIs with and without root contact were presented for each selected study. The mean differences between these recordings were calculated. These values were reported along with the 95 % confidence intervals. These effect measures were presented in a forest plot. Clinical and experimental studies were presented in separate figures. Statistical tests were carried out with Review Manager version 5.3 [63]. All intervention groups of multi-arm studies were listed in the table “Characteristics of included studies”. Unit of analysis issues could arise according to the level at which randomization occurs or in studies with repeated recordings of insertion torque values [64]. These issues were analysed for each specific study design, and our primary analysis was per randomized individual [43, 64].

**Fig. 3:**
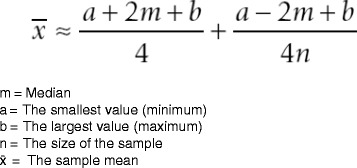
Formula for estimating the sample mean from the median, range, and the size of the sample [65]

**Fig. 4:**
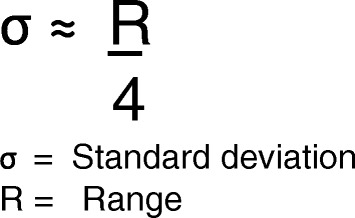
Formula for estimating the standard deviation from the range [65]

We did not foresee the poor reporting of various research data, which made it necessary to calculate a series of statistics. We adopted post hoc methods to extract missing research data and formulas to calculate pertinent statistics [65, 66]. These methods and formulas are widely accepted and are consistently used for this purpose in systematic reviews and meta-analyses. We used specific software to extract data from graphs and plots in the eligible studies and applied two formulas to calculate unreported statistics [65, 66]. We used the formula presented by Hozo et al. [65] to estimate the mean from the median, the ranges, and the sample size (Fig. 3). We used another formula by Hozo et al. [65] to convert ranges to standard deviations (Fig. 4). All methods to calculate unreported statistics are further explained in detail in Additional file 2.

### Synthesis of results

We presented and explained the characteristics and outcomes of the eligible studies in a narrative synthesis. This qualitative summary was conducted whether or not a quantitative data synthesis was considered appropriate. Characteristics of included studies were presented first and outcomes were subsequently listed according to the order of our research questions [43].

A meta-analysis would be conducted in the case of (1) low risk of bias in the selected studies, (2) consistent outcomes across the various studies, (3) low publication bias, (4) a high number of eligible studies, and (5) low heterogeneity [59, 64, 67]. Our planned methods for conducting meta-analyses were presented in our published protocol [63, 64, 67–76].

### Risk of bias across studies

#### Meta-biases

To assess the presence of reporting bias, we assessed whether protocols of trials were available and whether they were published prior to recruiting participants [43]. The Clinical Trial Register at the International Clinical Trials Registry Platform of the World Health Organization was searched to identify such studies published after 1 July 2005 [77]. We evaluated whether outcomes that were planned in the protocols were actually reported on in the published studies. Selective reporting of outcomes in all the eligible studies was also assessed as well as bias as a result of the outcomes of smaller studies. Outcomes with or without data obtained from contacted authors were also compared. Funnel plots were not conducted to explore reporting bias because only five studies were eligible [52, 78]. All procedures to assess meta-biases were conducted by three review authors (RMR, LL, and LR).

### Additional analyses

#### Heterogeneity and subgroup and sensitivity analyses

Sources of heterogeneity and methods to investigate heterogeneity were described in our published protocol [11]. Heterogeneity between research models, i.e. clinical, animal, and cadaver studies, was not assessed because these models were analysed separately. Planned and unforeseen post hoc subgroup analyses and meta-regression were not undertaken to investigate statistical heterogeneity because the number of the included studies was small and additional divisions in subgroups were not possible [64]. Planned sensitivity analyses were also not conducted because of the small number of eligible studies.

### Confidence in cumulative estimate/assessment of the quality of evidence (GRADE)

Our first research question assessed whether OMIs with root contact had higher insertion torque values than those without this target condition. Because this question does not specifically address a health problem, it does not qualify for an assessment using the Grading of Recommendations Assessment, Development, and Evaluation (GRADE) approach [79–83].

## Results

### Study selection

#### Research question 1

**Fig. 5:**
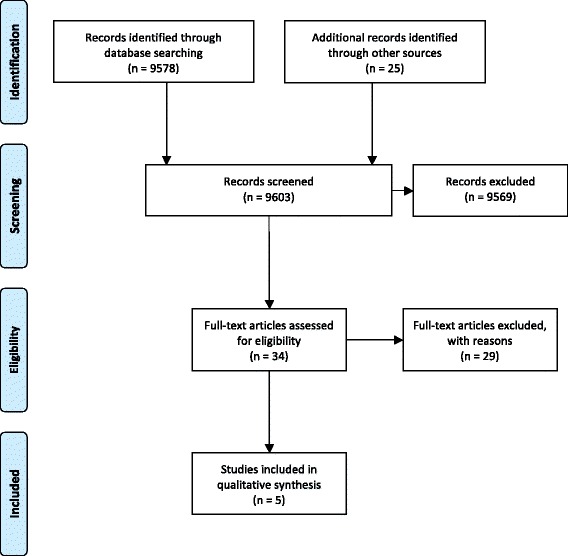
PRISMA flow diagram of the study selection procedures [44]

The study selection procedures for our first research question were presented in a PRISMA flow diagram (Fig. 5) [44]. The various search methods defined a total of 9603 abstracts with overlap. The total number of records retrieved for each data source and the search dates were presented in Additional file 3. The full texts of 34 articles were retrieved and were assessed for eligibility. Twenty-nine of these papers were subsequently excluded. The five eligible articles consisted of one human [6], two animal (dogs) [35, 84], and two cadaver (pigs) studies [32, 33]. One of these papers [32] was retrieved from the grey literature (Google Scholar B). The 29 excluded studies and the rationale for exclusion were presented in Additional file 4. Most studies were excluded because (1) insertion torque values were not recorded or because (2) insertion torque values were recorded, but an association with root vicinity was not assessed. All three reviewers were in complete agreement to exclude these studies.

#### Research question 2

No eligible studies were identified for the second research question.

#### Research question 3

Only one study [32] addressed the third research question.

### Study characteristics

The characteristics of the five eligible studies are summarized in Tables 1, 2, and 3. Additional data that were obtained through our procedures for ‘Contacting authors’ were not listed in these tables. An analysis of these methods and their outcomes are presented in the section ‘Contacting authors’. Table 1 presents the high heterogeneity in research models, the variation in treatment groups, and the underreporting of many items. Table 2 shows that four studies used digital torque tests and one study a mechanical index test. Time points for recording torque values varied widely between studies. Only one study measured insertion torque during the entire insertion path [32]. Brisceno et al. [25] inserted implants at different time points in a split mouth research model. Heterogeneity was also high for the types of ‘implants’, ‘location’, and ‘drilling technique’. Wilmes et al. [33] placed their screws at an inter-implant distance of 4 mm. This dimension is much smaller than the minimum recommended inter-implant distance of 5 × the diameter of the implant (5 × 1.3 mm), according to the standard of ASTM International [34]. Several studies used the pre-drilling technique [6, 33, 84]. Pre-drilling of pilot holes not only lowers the insertion torque values, but can also direct the implant into the root. Positivity test thresholds were not reported in any of the eligible studies. Table 3 shows that most studies used either two- or three-dimensional radiographs as the reference standard. Heterogeneous definitions of the target condition were reported between studies. The time points of conducting the reference standard also varied.

**Table 1:** Characteristics of included studies

Authors	Participants type, number, age, and sex	Research design and compared treatment groups	Consecutively treated participants	Power calculation
Motoyoshi et al. [6]	Self-drilling group 13 males and 28 females age 22.3 ± 7.9 years	Non-randomized study Self-drilling group versus Pre-drilling group	Not reported	Not reported
	Pre-drilling group 11 males and 27 females age 23.6 ± 8.1 years			
Chen et al. [84]	6 mongrel dogs 13–15 months	Split mouth design	Yes	Not reported
	Sex was not reported	Semi mandibles with root contact versus Semi mandible without root contact		
Brisceno et al. [25]	7 male Beagle dogs 20–24 months old	Split mouth design 6 weeks of healing after root contact versus 12 weeks of healing after root contact	Yes	Not reported
Wilmes et al. [33]	11 pig cadaver mandibles Age, and sex were not reported	Non-randomized study Random insertion of implants	Not applicable	Not reported
McEwan [32]	Pig cadaver mandibles Number, age, and sex were not reported	Non-randomized study Implants with root contact versus Implants without root contact versus Implants with root penetration	Not applicable	Yes

All data in this table represent those reported in the original manuscript. Additional data obtained through our protocol for ‘Contacting authors’ are not included in this table

**Table 2:** Index test-related domains

Authors	Index test	Time point of torque recording	Implant type, number, and dimensions	Location of insertion	Drilling technique	Test threshold
Motoyoshi et al. [6]	Digital	Terminal rotation of the screw	143 ISA^a^ 1.6 × 8 mm	Between maxillary first molar and second bicuspid	Self-drilling and pre-drilling	Not reported
Chen et al. [84]	Mechanical	During the last 1/3 of the insertion process	72 Leibinger^a^ 2.0 × 11 mm	With root contact Distal aspect of the first, second, third, and fourth mandibular premolars	Pre-drilling	Not reported
				Without root contact Under the bifurcation of the second, third, and fourth mandibular premolars and first molars		
Brisceno et al. [25]	Digital	Not reported	56 IMTEC^a^ 1.8 × 8 mm	Distal or mesial roots of the mandibular second, third, fourth premolars, and first molars	Self-drilling	Not reported
Wilmes et al. [33]	Digital	Last 0.2 mm of the insertion process	320 dual top^a^ 1.6 × 8 mm	Randomly in the mandibular alveolar ridge	Pre-drilling	Not reported
McEwan [32]	Digital	After 0.5 min of insertion and during the entire insertion process	30 3M^a^ 1.8 × 6 mm 30 Tomas^b^ 1.6 × 6 mm	Between or in contact with the mandibular first, second, and third premolars	Self-drilling	Not reported

^a^Implant types: ISA, Biodent (Tokyo, Japan); Leibinger (Freiburg, Germany); IMTEC (Ardmore Oklahoma); Dual top, Jeil Medical (Seoul, Korea); 3M TAD, Unitek™ (Monrovia, CA, USA); Tomas® pin, Dentaurum (Ispringen, Germany)

^b^All data in this table represent those reported in the original manuscript. Additional data obtained through our protocol for ‘Contacting authors’ are not included in this table

**Table 3:** Reference standard related domains

Authors	Reference standard	Target condition	Time point of conducting the reference standard
Motoyoshi et al. [6]	Three-dimensional cone beam computed tomography	No root contact One point contact 2 or more points of contact	After the application of an orthodontic force of 2 Newton
Chen et al. [84]	Two-dimensional radiographs and histology	Contacting or damaging the root surface	3, 12, or 24 weeks (depending on the subgroup) after conducting the index test
Brisceno et al. [25]	Two-dimensional peri-apical radiographs	Damaging the root	Immediately after conducting the index test
Wilmes et al. [33]	Digital scanning of histological slides	No root contact Unilateral root contact Bilateral root contact, i.e. penetration	After the preparation of histological slides
McEwan [32]	Three-dimensional cone beam computed tomography	No root contact Root contact Root penetration	Immediately after conducting the index test

All data in this table represent those reported in the original manuscript. Additional data obtained through our protocol for ‘Contacting authors’ are not included in this table

### Risk of bias in individual studies

All eligible studies in this systematic review were classified as ‘non-randomized’ because they either did not use [6, 32, 33] or did not clearly report methods of randomization [84] or this procedure was conducted for other outcomes [25] than those selected for our research questions [51, 85, 86]. We therefore adopted A Cochrane Risk Of Bias Assessment Tool for Non-Randomized Studies of Interventions (ACROBAT-NRSI) [46]. We applied this instrument to the original manuscripts of the five eligible studies.

The ACROBAT-NRSI scores for each of the seven domains of this tool were presented in Table 4.

**Table 4:** ACROBAT-NRSI risk of bias assessment [63]

Domain	Risk of bias in study by Motoyoshi et al. [6]	Risk of bias in study by Chen et al. [84]	Risk of bias in study by Brisceno et al. [25]	Risk of bias in study by Wilmes et al. [33]	Risk of bias in study by McEwan [32]
Bias due to confounding	Serious risk	Moderate risk	Serious risk	Moderate risk	Moderate risk
Bias in selection of participants into the study	No information	Low risk	Low risk	Low risk	Low risk
Bias in measurements of interventions	Moderate risk	Serious risk	Serious risk	Serious risk	Moderate risk
Bias due to departures from intended interventions	Low risk	Serious risk	Low risk	Low risk	Low risk
Bias due to missing data	Low risk	Low risk	Serious risk	Serious risk	Moderate risk
Bias in measurement of outcomes	Moderate risk	No information	No information	No information	No information
Bias in selection of the reported result	Low risk	Low risk	Low risk	Low risk	Low risk
Overall^a^	Serious	Serious risk	Serious risk	Serious risk	Moderate risk

Risk of bias scores

Low risk of bias: the study is comparable to a well-performed randomized trial with regard to this domain

Moderate risk of bias: the study is sound for a non-randomized study with regard to this domain but cannot be considered comparable to a well-performed randomized trial

Serious risk of bias: the study has some important problems in this domain

Critical risk of bias: the study is too problematic in this domain to provide any useful evidence on the effects of intervention

No information: no information on which to base a judgement about risk of bias for this domain

^a^Overall risk of bias score of the study. The overall risk of bias score is based on the severest risk of bias score that was identified for an individual domain; for example, when at least one domain was scored as ‘critical’ risk of bias, this means that the study as a whole has a risk of bias at least as severe

This table showed that all studies except the one by McEwan [32] scored at least one domain as ‘serious’ risk of bias. To maintain transparency of our bias ratings, we listed the rationales for these scores for each domain of each eligible study in an additional file (Additional file 5). In this document, we also presented lists of ‘preliminary consideration of confounders and co-interventions’. The consequences of additional information obtained from contacted authors on these bias assessments were presented in the section ‘Contacting authors’.

### Results of individual studies

#### Research question 1

The findings of the primary research studies are summarized in Table 5 and should be considered with the characteristics of the studies and the methodological quality in perspective. All eligible studies addressed our first research question, and a wide variation in insertion torque values between studies was recorded (Table 5). We contacted various authors to obtain additional research data to calculate pertinent statistics but were not always successful (see the section ‘Contacting authors’). We therefore used specific software to extract data from graphs and plots and applied two formulas to calculate unreported statistics (Additional file 2) [65, 66]. McEwan [32] did not list the standard deviations with the mean insertion torque values. WebPlotDigitizer was used to extract these measures from the insertion torque curves of each individual OMI [66]. For the selected study by Wilmes et al. [33], we used the formula presented by Hozo et al. [65] to estimate the mean from the median, the ranges, and the sample size (Fig. 3). For the study by Brisceno et al. [25], we used another formula by Hozo et al. [65] to convert ranges to standard deviations (Fig. 4).

**Table 5:** Insertion torque values in participants with or without implant-root contact

Authors	Model	Insertion torque values compared for different subgroups
Motoyoshi et al. [6]	Human patients	58 self-drilling without root contact: 7.1 ± 3.4 Ncm
		7 self-drilling 1 point contact: 8.7 ± 3.0 Ncm
		5 self-drilling multiple contacts: 8.1 ± 2.3 Ncm
		58 pre-drilling without root contact: 6.8 ± 2.3 Ncm
		7 pre-drilling 1 point contact: 7.4 ± 1.3 Ncm
		8 pre-drilling multiple contacts: 7.7 ± 2.1 Ncm
Chen et al. [84]	Mongrel dogs	25 pre-drilling without root contact 17.1 ± 5.9 Ncm
		47 pre-drilling with root contact 19.9 ± 6.6 Ncm
Brisceno^a^ [25]	Beagle dogs	*23* self-drilling without root contact 23.8 ± 3.6 Ncm
		*23* self-drilling with root contact 50.7 ± 7.2 Ncm
Wilmes et al. (a) [33]	Mandibles of pig cadavers	147 pre-drilling without root contact: mean 16.6 ± 5.7 Ncm
		50 pre-drilling with root contact: mean 18.5 ± 5.8 Ncm
Wilmes et al. (b) [33]	Mandibles of pig cadavers	147 pre-drilling without root contact: mean 16.6 ± 5.7 Ncm
		108 pre-drilling with root penetration: mean 21.9 ± 5.6 Ncm
McEwan (a) [32]	Mandibles of pig cadavers	3M implants
		10 self-drilling without root contact: mean 11.71 ± 0.9 Ncm
		10 self-drilling with root contact: mean 17 ± 2.5 Ncm
		10 self-drilling implants with root penetration were excluded because they did not further advance after root contact
McEwan (b) [32]	Mandibles of pig cadavers	Tomas implants
		10 self-drilling without root contact: mean 8.76 ± 0.8 Ncm
		7 self-drilling with root contact: mean 12.86 ± 1.2 cm
		10 self-drilling implants with root penetration were excluded because they did not further advance after root contact

Data obtained from the original manuscript are presented in black-type face print

Data obtained through our contacting author protocol are italicized

^a^The reference author, Dr. PH Buschang, was contacted and reported that 23 implants were placed with root contact and 23 without. This information was inserted in Table 5, but does not completely explain what happened to 5 of the 56 inserted implants because in the original manuscript, only a loss of five (fractured) implants was reported

According to protocol, each experimental model, i.e. human, dog, and cadaver was presented separately in a forest plot and the ‘mean difference’ (MD) in insertion torque values between implants with and without root contact was calculated with RevMan 5.3 [63] for each pre-established subgroup (Tables 6, 7, and 8). The high heterogeneity between studies explained the inconsistencies in effect sizes. Meta-analyses were not undertaken because of the serious heterogeneity and risk of bias issues. Unit of analysis issues (multiple implants per participant) could be a problem in the eligible studies [64, 87]. The impact of these issues could not be analysed in these studies because (1) the number of implants per participant was not reported [6, 32, 33] or (2) this statistic could not be calculated because the number of fractured implants or missing data per specific participant was not reported [25, 32] or (3) implant-root contact per specific participant was not reported [6, 25, 32, 33, 84]. None of the included studies made repeated recordings of insertion torque values. All studies and subgroups demonstrated higher insertion torque values for OMIs with the target condition than those without. These differences were not significant in any of the subgroups of the clinical study by Motoyoshi et al. [6], but sample sizes were small (Table 6). Significant mean differences in torque values were found in a study on beagle mandibles by Brisceno et al. [25] (MD, 4.64; 95 % CI, 3.50 to 5.79) and two cadaver subgroups by McEwan [32] (MD, 2.70; 95 % CI, 1.42 to 3.98) (MD, 3.97; 95 % CI, 2.17 to 5.78) and one cadaver subgroup by Wilmes et al. [33] (MD, 0.93; 95 % CI, 0.67 to 1.20). (Tables 7 and 8). Highest mean differences were found in the study by Brisceno et al. [25], which represented a 113 % increase in insertion torque values. Higher mean insertion torque differences were identified in the self-drilling groups compared with the pre-drilling groups in both the dog and cadaver models [25, 32, 33]. However, comparing these outcomes should be done with caution because of the heterogeneity between research models and study designs.

**Table 6:**
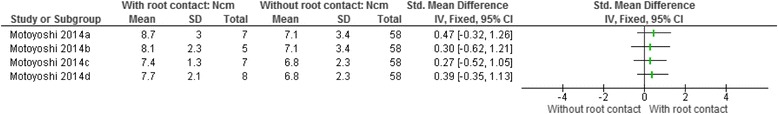
Insertion torque values in the clinical model in participants with or without implant-root contact

Characteristics of study or subgroup

Motoyoshi (a) [6]: self-drilling insertion with 1 point implant-root contact versus without implant-root contact

Motoyoshi (b) [6]: self-drilling insertion with multiple implant-root contacts versus without implant-root contact

Motoyoshi (c) [6]: pre-drilling insertion with 1 point implant-root contact versus without implant-root contact

Motoyoshi (d) [6]: pre-drilling insertion with multiple implant-root contacts versus without implant-root contact

**Table 7:**
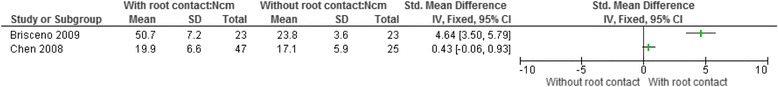
Insertion torque values in the dog model in participants with or without implant-root contact

Characteristics of study

Brisceno et al. [25]: self-drilling insertion with implant-root contact (damaging the root) versus without implant-root contact

Chen et al. [84]: pre-drilling insertion with implant-root contact (contacting or damaging the root) versus without implant-root contact

**Table 8:**
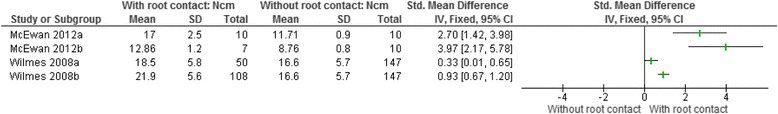
Insertion torque values in the pig cadaver model in participants with or without implant-root contact

Characteristics of study or subgroup

McEwan (a) [32]: self-drilling insertion with 3 M implants with implant-root contact versus without implant-root contact

McEwan (b) [32]: self-drilling insertion with Tomas implants with implant-root contact versus without implant-root contact

Wilmes et al. (a) [33]: pre-drilling insertion with implant-root contact versus without implant-root contact

Wilmes et al. (b) [33]: pre-drilling insertion with implant-root penetration versus without implant-root contact

#### Research question 2

Test positivity thresholds and the respective number of tests positives and test negatives were not reported in any of the studies (Table 2). Diagnostic accuracy statistics for our second research question could therefore not be calculated.

#### Research question 3

McEwan was the only researcher that measured torque during the entire insertion process and was therefore able to address the third research question [32]. This continuous recording provoked an ‘intermediate’ sudden increase of torque values at the moment of root contact and changed the more linear insertion graph to an upward angled curve. Differences in insertion graphics between implants with or without root contact were evident in this study [32]. McEwan also reported that directly hitting the root with OMIs with the self-drilling technique was impossible because implants did not further advance after making root contact and insertion torque values subsequently decreased to a lower plateau [32]. He excluded this subgroup from his statistical analysis. McEwan was the only author that reported this phenomenon.

#### Adverse effects

Three eligible studies presented adverse effects. Brisceno et al. [25] recorded that 5 of 56 implants fractured as a result of excessive insertion torque. Implants were subsequently inserted at torque values <55 Ncm [25]. Three Tomas pins fractured when contacting the root in McEwan’s study [32]. Chen et al. [84] showed the deficiency of two-dimensional radiographs as a reference standard because histology was necessary to accurately diagnose the target condition in 32 of the 72 implants.

### Risk of bias across studies

A protocol of any of the eligible studies was not identified in the literature or at The Clinical Trial Register at the International Clinical Trials Registry Platform of the World Health Organization [43, 77]. Exploring reporting bias with funnel plots was not indicated because of the small number of included studies [52, 78].

### Quality of the evidence

The quality of evidence according to the GRADE approach was not assessed because our first research question did not qualify for such an assessment, and for our second question, no eligible studies were identified [79].

### Contacting authors

Emails were sent to authors to obtain additional research data. Exemplary emails were presented in Additional file 6. Numerous contacting attempts were necessary to obtain a final reply and answers were often unsatisfactory (Table 9). However, for one study, we were able to obtain essential data to calculate most of the necessary statistics [25]. Without this information, this study would have been excluded from our review. Additional data from contacted authors were also important to reconsider or confirm risk of bias assessments (Table 9) [25, 32, 84]. Several risk of bias scores were either upgraded or downgraded as a result of our contacting procedures (Table 9). A detailed report of the outcomes and the difficulty of the contacting procedures were presented in Additional file 7.

**Table 9:** Outcomes and consequences of contacting authors of eligible studies

Author	Number of contacting attempts^a^	Willingness of authors to reply	Number of research questions answered	Additional research data provided by the contacted authors and its consequences
Motoyoshi et al. [6]	5 attempts	Unclear	0 of 6 questions	• No additional research data were provided. • No consequences for the risk of bias scores were therefore assigned.
Chen et al. [84]	3 attempts	Yes	1 of 1 question	• Outcome assessors were blinded. This information changed the risk of bias score for the domain ‘Bias in measurement of outcomes’ from ‘No information’ to ‘Low’ risk of bias.
Brisceno et al. [25]	7 attempts	Yes	6 of 6 questions	• Insertion torque was measured at complete insertion of the 8 mm implant length. This information was not sufficient to lower the risk of bias score for the domain ‘Bias in measurements of interventions’. • Personnel and outcome assessors were not blinded. This information changed the risk of bias score for the domain ‘Bias in measurement of outcomes’ from ‘No information’ to ‘Serious’ risk of bias. • The sample consisted of 23 implants with and 23 without root contact. This information changed the risk of bias score for the domain ‘Bias due to missing data’ from ‘Serious’ risk to ‘Moderate’ risk of bias. This information also permitted the calculation of various statistics and list them in a forest plot.
Wilmes et al. [33]	5 attempts	Yes	2 of 6 questions	• Animals were 8–10 months old. Most of our questions were not answered by the contacted authors and no consequences were therefore applied.
McEwan [32]	2 attempts	Yes	7 of 7 questions	• Animals were approximately the same age. Different screw types were randomly assigned to the mandibles. This information changed the risk of bias score for the domain ‘Bias due to confounding’ from ‘Moderate’ to ‘Low’ risk of bias. • Outcome assessors and personnel were not blinded. This information changed the risk of bias score for the domain ‘Bias in measurement of outcomes’ from ‘No information’ to ‘Serious’ risk of bias.

^a^This number refers to the total number of attempts by email to get an answer from a contacted author

This number also includes the number of attempts to contact a co-author(s). An initial attempt or a subsequent reminder attempt was each counted as one attempt. As soon as authors replied, successive emails were not counted as additional attempts. Ideally, only two attempts are made: (1) the email to request the ‘willingness to reply’ and (2) the email to get additional data from the contacted authors

Attempts of sending emails from other email addresses were not counted as additional attempts. Sending such emails could at times be indicated because our initial email could be identified as ‘spam mail’ and could then be deleted by the receiving internet provider

## Discussion

### Summary of main results

One clinical, two animal, and two cadaver studies were identified as eligible for our first research question. These studies reported the necessary data to calculate the statistics for this question. This same data set in combination with a test threshold for specific insertion torque values would have been sufficient to also answer our second research question, but none of these studies defined such a threshold. As a consequence test positives and negatives were not scored and important research information was wasted. No additional studies were identified that addressed our second research question.

Insertion torque values of implants with root contact were higher than those without in all five eligible studies. These differences were significant in one animal study [25] and in three cadaver subgroups [32, 33], but not in the clinical study by Motoyoshi et al. [6] (Tables 6, 7, and 8). However, the subsets of patients with the target condition in this latter study were small [6]. Torque differences were higher in the self-drilling compared with the pre-drilling surgical technique in the animal and cadaver models. Measuring insertion torque during the entire insertion process could have clinical utility to get immediate information when the implant makes root contact, but this evidence came only from one cadaver study [32].

The validity of most of these outcomes should be carefully weighed because differences in research models, underreporting, and the methodological quality were all major issues in the selected articles (Table 4). Adverse effects referred to fractured implants and were only recorded in the self-drilling groups [25, 32]. Reporting of adverse effects was in general suboptimal.

### Strengths and weaknesses of the systematic review

The strengths of this systematic review included: (1) it was first published as a protocol [11]; (2) it was based on a broad-spectrum literature search using numerous databases and other search methods [51]; (3) it was conducted by experienced reviewers, topic experts, and evidence-based healthcare methodologists who have produced several systematic reviews on OMIs [55–57]; and (4) it incorporated non-randomized studies, animal, and cadaver models [88–93]. The inclusion of animal studies and cadaver models in a systematic review is important because (1) they might provide additional information on the usefulness of conducting the index test; (2) they could provide information on how to design future research studies on our clinical question; (3) considering outcomes from animal studies avoids wasting valuable research information, financial resources, and duplication [88, 89]. The importance of these issues was further stressed by Iain Chalmers, one of the founders of the Cochrane Collaboration, in a recent international symposium on systematic reviews in laboratory animal science [90]; and (4) not considering these studies would risk that knowledge creation on this topic would come to a standstill. These issues are further strengthened in the context of the high prevalence of the target condition, the risk of biologic damage of the interventional procedure, the instability of implants with the target condition, and the underreporting of adverse effects of interventions [8–10, 13, 94, 95]. It could also be considered unethical not to include experimental studies when only limited numbers of clinical studies are identified. This issue should be further considered in the context that harms of interventions are generally poorly reported [95].

Weaknesses of the review included: (1) the inclusion of different research models and the heterogeneity of the selected studies could have introduced applicability issues; (2) serious risk of bias scores for most eligible studies; (3) the underreporting of many key items in most selected studies; and (4) the poor responding of several contacted authors.

Numerous sources of heterogeneity were identified in the eligible studies (Tables 1, 2, 3, 4, 5, 6, 7, and 8) [96]. Heterogeneity in a systematic review should be expected and is not a weakness itself, but it could create applicability issues [73]. In addition, the quality of the research findings of most identified studies was conditioned by serious risks of bias (Table 4). Synthesizing the outcomes of these heterogeneous and biased studies in a meta-analysis was therefore not indicated, and findings were discussed in a narrative format (Tables 6, 7, and 8) [74]. To avoid misleading the reader, we did not present the summary diamonds of the effect estimates in the respective forest plots.

Poor reporting was evident in all studies, which made it difficult to detect differences between studies [97]. However, one should consider that our research questions were not always the primary objectives of our eligible studies, and poor reporting of secondary outcomes could have been the consequence. To avoid introducing imprecision, we did not use ‘Not reporting’ as a criterion to increase risk of bias scores. Nevertheless, all studies except one [32] scored serious risk of bias scores for at least one domain of the ACROBAT-NRSI tool [46]. These issues are addressed in the next section.

Obtaining a response from authors to our requests for additional information was often difficult. This is unfortunate because sharing research data promotes scientific integrity and will reduce research waste [98]. However, responses to our research questions had a positive effect on study selection because one additional study [25] became eligible for inclusion and strengthened the validity of the outcomes. Responses also helped to fine-tune risk of bias assessments (Table 9) [25, 32, 33, 84]. Although response rates of contacted authors were suboptimal, they were much better than those obtained in our previous systematic reviews on OMIs [55, 56]. Having attached the published protocol of our systematic review to our contacting email could perhaps have accounted for the improved response rate.

### Applicability of findings to clinical practice

Notwithstanding the limitations of the eligible primary studies, many of the identified issues could have an impact on the clinical utility of torque recordings and are important when designing new research studies on this topic. These items are discussed using the domains of the ACROBAT-NRSI tool as a framework [46].

### Bias due to confounding and bias in selection of participants into the study

Confounding and selection procedures of participants were sources of bias in most eligible studies (Tables 1 and 4). Randomizing patients to treatments with or without root contact is not feasible for ethical reasons and future research studies should therefore be designed as prospective cohorts on consecutively treated human participants. A prevalence of 20 % of the occurrence of the target condition and a 25 % increase in insertion torque values as the hypothetical starting threshold for test positivity could be considered when conducting power calculations for our second research question. The validity of this latter threshold is unknown because only limited information has been published on this measure [6, 25]. The pitfalls of the split mouth design should also be considered because variations in insertion torque levels could be the result of this study design when experiments are conducted at different time points [99].

### Bias in measurements of interventions: the index test

For the pre-drilling technique, pilot holes are drilled for the entire length of the screw. This procedure reduces the resistance in the bone when implants are inserted. When the tip of the pilot drill touches the root, it could remove part of the root surface which further reduces this resistance. This phenomenon could explain why increases in torque values upon root contact were smallest in most pre-drilling groups (Tables 6, 7, and 8) [6, 33, 84]. This decrease in the severity of the target condition can deflate sensitivity and inflate specificity. The self-drilling technique is probably more accurate in diagnosing implant-root contact and probably also produces less root damage than the pre-drilling technique. McEwan [32] showed that OMIs did not advance at all after direct root contact using the self-drilling technique. These factors could be two important rationales for avoiding pre-drilling when conducting the index test in clinical practice. Similar findings were also reported for screws placed between dental roots for intermaxillary fixation in maxillofacial surgery [28]. Widar et al. [28] reported that injury to the dental roots was only found in the pre-drilled group and not in the self-drilled screws. However, our reasoning should be placed in the perspective that fractured implants were only identified in the eligible studies that used the self-drilling technique [25, 32]. OMIs should therefore be inserted with great prudence below their peak torque levels of fracture when using the self-drilling technique [25, 100].

In our eligibility criteria, we aimed at ‘The recording of insertion torque values during the insertion of OMIs’ [11]. This broad spectrum criterion was chosen in order to avoid excluding pertinent studies. Previous systematic reviews on similar topics have shown that definitions of the time points for recording torque values are often broad and that subsequent analyses can filter out differences between subgroups [55, 56]. One of these reviews has also explained the importance of recording insertion torque not just at a specific time point, but in the perspective of four additional insertion parameters: (1) the total number of rotations necessary to insert a screw for its entire thread length; (2) the number of rotations of the implant at the moment of the torque recording; (3) the axial load; and (4) the insertion depth [56]. Measuring the index test remains imprecise without this information because slippage and stalling as a result of root contact or insufficient axial loading can influence insertion depth, the number of rotations, or the time necessary to advance to a certain depth. None of the eligible studies assessed these four insertion parameters in combination with the recordings of insertion torque values. The trustworthiness of these measures could therefore be jeopardized. These issues should be considered when developing protocols for future research studies. A torque sensor that measures torque as a function of the number of rotations could be a starting point.

**Fig. 6:**
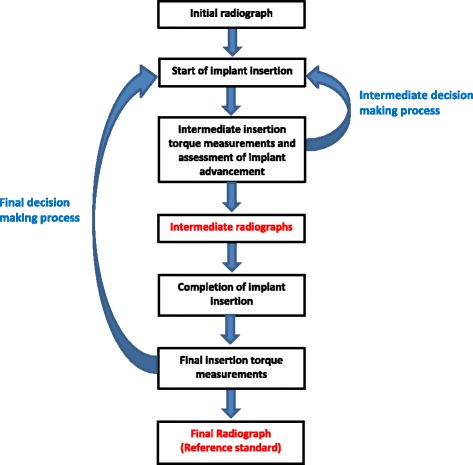
Potential new diagnostic pathway for assessing implant-root contact. The steps in red-type face can be eliminated when the index can be used for intermediate decision making and is more accurate than the reference standard

Even better could be the measuring of torque during the entire insertion path [32]. McEwan demonstrated the change in the insertion graph when the implant touched the root [32]. These measures can only be obtained with digital torque recorders and provide valuable information on early root contact or on stripping or stalling. Such recordings can be helpful for intermediate decision-making on whether to stop or redirect implant insertion. Incorporating continuous torque recordings with digital sensors could have an important impact on clinical practice and future research studies. The validity of a new diagnostic pathway that includes intermediate decision making is depicted in Fig. 6 and should be tested in such studies. We also incorporated the intermediate assessment of implant advancement in relation to the number of rotations of the screws because McEwan showed that OMIs did not advance at all after direct root contact [32].

This systematic review also pointed at the importance of standardizing implant-, operator-, surgery-, and location-related factors prior to conducting studies on our research questions. These issues have been addressed by ASTM International, which has established a standard (ASTM Standard F543-07^ε1^) [34] for conducting insertion torque tests in artificial bone. This guideline has set the minimum inter-implant distance at 5 × the diameter of the implant to avoid weakening of the bone as a result of previous insertions of implants in neighbouring sites. The small inter-implant space of 4 mm, instead of a minimal distance of 6.5 mm (5 × 1.3 mm), could explain the wide variance in effect estimates in the cadaver study by Wilmes et al. [33].

### Bias in measurements of interventions: the reference standard and the target condition

In one eligible study, 32 of 72 two-dimensional radiographs could not adequately diagnose the target condition [84]. Future studies should use three-dimensional radiographs as the reference standard because they are more accurate than two-dimensional imaging techniques [101, 102]. This advantage should be weighed in the context of the higher radiation exposure for the 3D imaging compared with the conventional 2D dental radiographs [103]. Clear definitions of the target condition were presented in three of the eligible studies [6, 32, 33]. Future studies should follow this example because different effect estimates were recorded for different types of target conditions.

Motoyoshi et al. [6] loaded the OMIs with orthodontic forces prior to conducting the reference standard, which could have contaminated the radiograph by the superimposition of orthodontic coils springs or by displacing the implants. This latter scenario can cause an over- or underestimation of the proportion of patients with a target condition, i.e. introducing respectively disease progression or recovery bias [104]. Chen et al. [84] took the reference standard at different time points, which could also have led to systematic error.

### Bias due to departures from intended interventions and bias due to missing data

Bias due to departures from intended interventions was only identified in one study where histology was necessary because the two-dimensional radiographs could not adequately diagnose the target condition [84]. Poor reporting made it impossible to draw the flow of participants in two studies [25, 33]. In one study [33], 15 implants were not accounted for and another article [25] did not report the number of implants with or without root contact, but these numbers were finally obtained through contacting the authors. However, what happened to some of the 56 inserted implants was still not explained. Ignoring drop-outs raises the risk of introducing bias [59, 87].

### Bias in measurement of outcomes and bias in selection of the reported result

Bias in the measurement of outcomes was a concern in all studies, mostly as a result of underreporting (Table 4). Calibration and blinding of outcome assessors and the inclusion of more than just one of these operators are important variables for the reduction of bias in this domain. All studies scored ‘low risk’ of bias in the selection of the reported result (Table 4).

### Suggested next steps

The outcomes of this systematic review should be considered in the perspective of the different research models, heterogeneity, quality, and reporting issues. The implementation of the index test under review as a replacement or triage test should be avoided because our diagnostic accuracy question was not addressed by any of the eligible studies. Variables discussed in the previous section and those that are illustrated in Fig. 6 should be considered with prudence when inserting OMIs. Using a digital torque sensor as an add-on test is possible because of its shared use for the assessment of implant fractures and implant stability, its minimal costs, and the absence of adverse effects. Transparent reporting on both the desired and adverse effects of implant insertion is an essential component of this new diagnostic pathway.

Because of these characteristics and because the target condition is created during the index test procedures, it will be possible to address a variety of research questions from the same data set. Answers should be first sought in cadaver and in vivo animal studies. A detailed transparent protocol has to be developed for this purpose that includes input from (1) the items presented in our ‘Discussion’ section of the QUADAS-2 tool; (2) the figure for the potential new diagnostic pathway for assessing implant-root contact (Fig. 6); (3) the STARD checklist [53]; (4) ASTM International [34]; (5) our protocol with the tailored QUADAS-2 tool [60]; and (6) a variety of stakeholders, e.g. patients, clinicians, researchers, the paying party, and manufacturers [54, 62]. Measuring insertion torque during the entire insertion process and the reporting of adverse effects of interventions should become key elements of such protocols [32].

## Conclusions

- All eligible studies addressed our first research question, but none investigated the second question. This research waste could have been avoided because the answers to both questions could have been extracted from the same data set.
- Torque levels of OMIs inserted with root contact were higher than those without in all research models. Highest torque differences were identified in the self-drilling compared with the pre-drilling groups in the animal and cadaver models. One study [32] showed the importance of continuous recording of torque values during the entire implant insertion process. However, findings should be considered in the context of the different research models, the high heterogeneity, and the serious risk of bias issues.
- This research study demonstrated the importance of including non-randomized studies and animal and cadaver models in a systematic review because they (1) were essential for this research topic in the context of its ethical constraints and (2) permitted the uncovering of both expected and unexpected variables associated with the accuracy of the index test. Not including such studies could have slowed down knowledge creation on this topic.
- A new diagnostic pathway for the index test was proposed for future research studies, i.e. torque and a variety of other parameters are recorded during the entire self-drilling insertion process, which could provide immediate information when the implant makes root contact [32]. Transparent reporting of both the desired and undesired effects of the insertion process is a key component of this new diagnostic pathway.
- This systematic review also showed that responses of contacted authors were helpful, but often difficult to obtain. Not replying to systematic reviewers does not only complicate the work of these researcher, but could also waste potential valuable information [85, 88].

## Additional files

Additional file 1: PRISMA 2009 Checklist. (DOC 64 kb) Additional file 2: Calculating missing statistics. (DOCX 21 kb) Additional file 3: Records per data source. (DOCX 16 kb) Additional file 4: Excluded full text articles. (DOCX 26 kb) Additional file 5: Rationales for the ACROBAT-NRSI risk of bias scores. (DOCX 29 kb) Additional file 6: Exemplary emails for contacting authors. (DOCX 18 kb) Additional file 7: Outcomes of contacting authors. (DOCX 16 kb)

## Abbreviations

Ncm: Newton centimetre
OMIs: orthodontic mini-implants
