# Supplementary material for: Insertion torque recordings for the diagnosis of contact between orthodontic mini-implants and dental roots: a systematic review
Source: Syst Rev. 2016 Mar 31;5:50. doi: 10.1186/s13643-016-0227-3 (PMC4818448; doi:10.1186/s13643-016-0227-3)
Supplement: Additional file 2: — Calculating missing statistics. (DOCX 21 kb) [file 13643_2016_227_MOESM2_ESM.docx]

**Additional file 2. Calculating missing statistics**

**Extracting data from plots and graphs**

Specific software was used to extract data from plots and graphs. McEwan [32] did not list the standard deviations with the mean insertion torque values. WebPlotDigitizer was used to extract these measures from the insertion torque curves of each individual OMI [66]. For the eligible study by Wilmes et al. [33]we also used WebPlotDigitizer software to extract torque ranges from the Box-Whisker plots [66].

**Converting medians to means**

Wilmes and co-workers [33] reported the median and not the mean and ranges of their torque recordings in their research study. We used WebPlotDigitizer software [66] to extract torque ranges from the Box-Whisker plots in this study and applied a formula by Hozo and co-workers [65] to estimate the mean from the median and the ranges and the sample size. This formula and the calculated statistics are respectively presented in figure 1 and table 1 of this additional file. The original outcome measure Newton millimeter (Nmm) in the study of Wilmes et al. [33] was subsequently modified to the outcome measure Newton centimeter (Ncm) of this systematic review.

**Figure 1. Formula for estimating the sample mean from the median, range, and the size of the sample** [65]


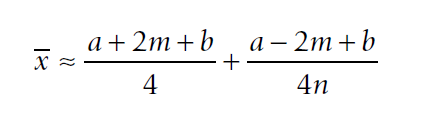


m = Median

a = The smallest value (minimum)

b = The largest value (maximum)

n = The size of the sample

x̄ = The sample mean

**Table 1. Various statistics for the different target conditions in the study by Wilmes et al. [33]**

| **Target condition** | **Sample size** | **Median (Nmm)** | **Mean (Nmm)** | **Standard deviations (Nmm)** | **Range (Nmm)** |
| --- | --- | --- | --- | --- | --- |
| No implant-root contact | 147 | 161 | 166,3 | 57 | 32-311 |
| Implant-root contact | 50 | 185 | 184,7 | 58 | 57-312 |
| Root penetration | 108 | 215 | 218,8 | 56 | 99-346 |

**Converting ranges to standard deviations**

In the eligible study by Brisceno et al. [25], the range, but not the standard deviation of insertion torque values of implants with and without root contact was reported. We calculated the standard deviation from the range according to a formula presented by Hozo et al. [65](figure 2). This formula is used for sample sizes between 16 and 70 with normally distributed data [65]. The calculated statistics are presented in table 2.

**Figure 2. Formula for estimating the standard deviation from the range [65]**

σ ≈ R

4

σ = Standard deviation

R = Range

**Table 2. Various statistics for the different target conditions in the study by Brisceno et al. [25]**

| **Target condition** | **Sample size** | **Mean (Ncm)** | **Standard deviation (Ncm)** | **Range (Ncm)** |
| --- | --- | --- | --- | --- |
| No implant-root contact | 23 | 23.8 | 3.6 | 16.6-31 |
| Implant-root contact | 23 | 50.7 | 7.2 | 36.4-65.2 |
