# Supplementary material for: Insertion torque recordings for the diagnosis of contact between orthodontic mini-implants and dental roots: a systematic review
Source: Syst Rev. 2016 Mar 31;5:50. doi: 10.1186/s13643-016-0227-3 (PMC4818448; doi:10.1186/s13643-016-0227-3)
Supplement: Additional file 3: — Records per data source. (DOCX 16 kb) [file 13643_2016_227_MOESM3_ESM.docx]

**Additional file 3. Records per data source**

**Search terms for the index test** [11]

Torque OR insertion torque OR torqueing OR torqueing OR torque sensor OR torque device OR torquing device OR torqueing device OR torque screwdriver OR torque driver

**Search terms for the target condition (1)** [11]

Target condition (1) Root OR root contact OR root vicinity OR dental root OR root damage OR tooth OR teeth OR tooth contact OR tooth vicinity

**Search terms for the target condition (2)** [11]

Implant OR mini implant OR micro implant OR microimplant OR screw OR mini screw OR miniscrew OR micro screw OR microscrew OR temporary anchorage device

**Abstracts retrieved by electronic searching, hand searching, and reference searching * ****

| **Source of records** | **Date of search** | **Number of abstracts** |
| --- | --- | --- |
| Google Scholar Beta | 19-06-2015 | 5050 |
| PubMed (MEDLINE) | 19-06-2015 | 312 |
| EMBASE (Ovid) | Week 24 2015 | 154 |
| Science Direct | 19-06-2015 | 9 |
| Cochrane Central Register of Controlled Trials (CENTRAL) | 19-06-2015 | 36 |
| “Related Articles” in PubMed | 19-06-2015 | 125 |
| Turning research into practice (TRIP) Database | 19-06-2015 | 76 |
| NHS Evidence | 19-06-2015 | 37 |
| SUMSearch2 | 19-06-2015 | 294 |
| Science Citation Index | 19-06-2015 | 0 |
| Scopus | 19-06-2015 | 137 |
| Web of Science | 19-06-2015 | 1410 |
| African Index Medicus | 19-06-2015 | 0 |
| African Journals online (AJOL) | 19-06-2015 | 119 |
| Australasian Medical Index | 19-06-2015 | 24 |
| Index Medicus for the Eastern Mediterranean Region | 19-06-2015 | 0 |
| IndMED | 19-06-2015 | 259 |
| KoreaMed | 19-06-2015 | 10 |
| LILACS | 19-06-2015 | 21 |
| Index Medicus for the South-East Asia Region (IMSEAR) | 19-06-2015 | 0 |
| Western Pacific Region Index Medicus (WPRIM) | 19-06-2015 | 285 |
| Open Grey | 19-06-2015 | 548 |
| The Health Management Information Consortium (HMIC) | 19-06-2015 | 0 |
| The National Technical Information Service (NTIS) | 19-06-2015 | 19 |
| ProQuest Dissertations & Theses | 19-06-2015 | 1 |
| DissOnline | 19-06-2015 | 0 |
| Conference Proceedings Citation Index (Covered by Web of Science) | 19-06-2015 | No additional abstracts to those already presented under Web of Science |
| BIOSIS Citation Index | 19-06-2015 | No additional abstracts to those already presented under Web of Science |
| Meeting Abstracts | 19-06-2015 | 4 |
| ISI Proceedings |  | 3 |
| Database of Abstracts of Reviews of Effects (DARE) in the Centre for Reviews and Dissemination (CRD) Database | 19-06-2015 | 3 |
| Health Technology Assessment database (HTA) in the Centre for Reviews and Dissemination (CRD) Database | 19-06-2015 | 0 |
| MedlinePlus Guidelines | 19-06-2015 | 81 |
| Guidelines of the Australian National Health and Medical Research Council | 19-06-2015 | 473 |
| Guidelines of the Canadian Medical Association | 19-06-2015 | 0 |
| National Guideline Clearinghouse | 19-06-2015 | 12 |
| New Zealand Guidelines Group | 19-06-2015 | 3 |
| NICE Clinical Guidelines | 19-06-2015 | 8 |
| Citation alerts in Pubmed and Ovid | 19-06-2015 | 65 |
| Handsearching | 19-06-2015 | 7 |
| Reference lists | 19-06-2015 | 15 |
| Correspondence | 19-06-2015 | 3 |
|  |  |  |
| **Total** |  | 9603 abstracts with overlap |

* During the transfer of search terms, all search strategies were copied and pasted from the original

search strategy without re-typing, because this procedure can introduce errors [51]. To avoid the

inappropriate exclusion of pertinent articles, broader searches were conducted when no abstracts

were found with the reference search strategy, i.e., the search strategy used for Pubmed

(MEDLINE).

** For each individual search engine we applied the appropriate characters to truncate or explore

search terms.
