# Supplementary material for: Insertion torque recordings for the diagnosis of contact between orthodontic mini-implants and dental roots: a systematic review
Source: Syst Rev. 2016 Mar 31;5:50. doi: 10.1186/s13643-016-0227-3 (PMC4818448; doi:10.1186/s13643-016-0227-3)
Supplement: Additional file 4: — Excluded full text articles. (DOCX 26 kb) [file 13643_2016_227_MOESM4_ESM.docx]

**Additional file 4. Excluded full-text articles and references**

**Excluded full-text articles and reasons for exclusion (n=29)**

| **Authors** | **Reasons for exclusion*** |
| --- | --- |
| Ahmed V 2012 | A |
| Asscherickx 2005 | B |
| Arismendi 2007 | A |
| Asscherickx 2008 | B |
| Brisceno 2007 | C |
| Chaddad 2008 | A |
| Cho 2010 | B |
| Hembree 2009 | B |
| Holst 2010 | A |
| Huang 2012 | A |
| Iwai 2015 | B |
| Janson 2013 | B |
| Kang 2009 | B |
| Kau 2010 | B |
| Kim 2010 | B |
| Kim 2011 | B |
| McManus 2010 | A |
| Motoyoshi 2006 | A |
| Motoyoshi 2007a | A |
| Motoyoshi 2007b | A |
| Motoyoshi 2010 | A |
| Shigeeda 2014 | A |
| Shinohara 2013 | A |
| Son 2014 | A |
| Suzuki 2011 | A |
| Suzuki 2013 | A |
| Watanabe 2013 | B |
| Wilmes 2008 | A |
| Wilmes 2011 | A |

***Reasons for exclusion**

A: Insertion torque values were recorded, but an association with root vicinity was not assessed.

B: Insertion torque values were not recorded

C: Original MSc thesis of an included study by Brisceno et al. [25]

**References excluded full-text articles**

**Ahmed V 2012**

[Ahmed V KS](http://www.ncbi.nlm.nih.gov/pubmed/?term=Ahmed%20V%20KS%5BAuthor%5D&cauthor=true&cauthor_uid=22554748), [Rooban T](http://www.ncbi.nlm.nih.gov/pubmed/?term=Rooban%20T%5BAuthor%5D&cauthor=true&cauthor_uid=22554748), [Krishnaswamy NR](http://www.ncbi.nlm.nih.gov/pubmed/?term=Krishnaswamy%20NR%5BAuthor%5D&cauthor=true&cauthor_uid=22554748), [Mani K](http://www.ncbi.nlm.nih.gov/pubmed/?term=Mani%20K%5BAuthor%5D&cauthor=true&cauthor_uid=22554748), [Kalladka G](http://www.ncbi.nlm.nih.gov/pubmed/?term=Kalladka%20G%5BAuthor%5D&cauthor=true&cauthor_uid=22554748). Root damage and repair in patients with temporary skeletal anchorage devices. [Am J Orthod Dentofacial Orthop.](http://www.ncbi.nlm.nih.gov/pubmed/?term=root+damage+and+repair+in+patients+with+temporary+skeletal) 2012 May;141(5):547-55.

**Arismendi 2007**

Arismendi JA, Ocampo ZM, Morales M, Gonzalez FJ, Jaramillo PM, Sanchez A. Evaluation of stability of mini implants as bony anchorage for upper molar intrusion. Revista Facultad de Odontologia Universidad de Antioquia 2007;19:59-73.

**Asscherickx 2005**

Asscherickx K, Vannet BV, Wehrbein H, Sabzevar MM. [Root repair after injury from mini-screw.](http://www.ncbi.nlm.nih.gov/pubmed/16164464) Clin Oral Implants Res. 2005 Oct;16(5):575-8.

**Asscherickx 2008**

[Asscherickx K](http://www.ncbi.nlm.nih.gov/pubmed/?term=Asscherickx%20K%5BAuthor%5D&cauthor=true&cauthor_uid=18632839), [Vande Vannet B](http://www.ncbi.nlm.nih.gov/pubmed/?term=Vande%20Vannet%20B%5BAuthor%5D&cauthor=true&cauthor_uid=18632839), [Wehrbein H](http://www.ncbi.nlm.nih.gov/pubmed/?term=Wehrbein%20H%5BAuthor%5D&cauthor=true&cauthor_uid=18632839), [Sabzevar MM](http://www.ncbi.nlm.nih.gov/pubmed/?term=Sabzevar%20MM%5BAuthor%5D&cauthor=true&cauthor_uid=18632839). Success rate of miniscrews relative to their position to adjacent roots. [Eur J Orthod.](http://www.ncbi.nlm.nih.gov/pubmed/18632839) 2008 Aug;30(4):330-5.

**Brisceno 2007**

Healing of the root and surrounding structures following intentional damage with a miniscrew implant (MSI). MSc thesis. Submitted to the Office of Research and Graduate Studies of The Texas A&M University System Health Science Center in partial fulfillment of the requirements for the degree of Master of Science. Baylor College of Dentistry, Texas (USA), Department of Orthodontics; 2007.

**Chaddad 2008**

Chaddad K, Ferreira AF, Geurs N, Reddy MS. Influence of surface characteristics on survival rates of mini-implants. Angle Orthod 2008;78:107-13.

**Cho 2010**

[Cho UH](http://www.ncbi.nlm.nih.gov/pubmed/?term=Cho%20UH%5BAuthor%5D&cauthor=true&cauthor_uid=19852652), [Yu W](http://www.ncbi.nlm.nih.gov/pubmed/?term=Yu%20W%5BAuthor%5D&cauthor=true&cauthor_uid=19852652), [Kyung HM](http://www.ncbi.nlm.nih.gov/pubmed/?term=Kyung%20HM%5BAuthor%5D&cauthor=true&cauthor_uid=19852652). Root contact during drilling for microimplant placement. Affect of surgery site and operator expertise. [Angle Orthod.](http://www.ncbi.nlm.nih.gov/pubmed/?term=6.%09Root+contact+during+drilling+for+microimplant+placement+affect+of+surgery+site+andoperator+expertise.) 2010 Jan;80(1):130-6.

**Hembree 2009**

[Hembree M](http://www.ncbi.nlm.nih.gov/pubmed/?term=Hembree%20M%5BAuthor%5D&cauthor=true&cauthor_uid=19268823), [Buschang PH](http://www.ncbi.nlm.nih.gov/pubmed/?term=Buschang%20PH%5BAuthor%5D&cauthor=true&cauthor_uid=19268823), [Carrillo R](http://www.ncbi.nlm.nih.gov/pubmed/?term=Carrillo%20R%5BAuthor%5D&cauthor=true&cauthor_uid=19268823), [Spears R](http://www.ncbi.nlm.nih.gov/pubmed/?term=Spears%20R%5BAuthor%5D&cauthor=true&cauthor_uid=19268823), [Rossouw PE](http://www.ncbi.nlm.nih.gov/pubmed/?term=Rossouw%20PE%5BAuthor%5D&cauthor=true&cauthor_uid=19268823). Effects of intentional damage of the roots and surrounding structures with miniscrew implants. [Am J Orthod Dentofacial Orthop.](http://www.ncbi.nlm.nih.gov/pubmed/?term=hembree+effects+of+intentional+damage+of+the) 2009 Mar;135(3):280.e1-9; discussion 280-1.

**Holst 2010**

Holst AI, Karl M, Karolczak M, Goellner M, Holst S. [Quantitative assessment of orthodontic mini-implant displacement: the effect of initial force application.](http://www.ncbi.nlm.nih.gov/pubmed/19907734) Quintessence Int. 2010 Jan;41(1):59-66.

**Huang 2012**

Huang CT, Lai EHH, Chang HH, Chang BE, Chen YH, Wang YP, Chen YJ, Chang JZC, Yao CCJ. Damage to the root after tooth movement towards a temporary anchorage device: An animal pilot study. J Dent Sciences. 2012;7:171-178.

**Iwai 2015**

[Iwai H](http://www.ncbi.nlm.nih.gov/pubmed/?term=Iwai%20H%5BAuthor%5D&cauthor=true&cauthor_uid=25836008), [Motoyoshi M](http://www.ncbi.nlm.nih.gov/pubmed/?term=Motoyoshi%20M%5BAuthor%5D&cauthor=true&cauthor_uid=25836008), [Uchida Y](http://www.ncbi.nlm.nih.gov/pubmed/?term=Uchida%20Y%5BAuthor%5D&cauthor=true&cauthor_uid=25836008), [Matsuoka M](http://www.ncbi.nlm.nih.gov/pubmed/?term=Matsuoka%20M%5BAuthor%5D&cauthor=true&cauthor_uid=25836008), [Shimizu N](http://www.ncbi.nlm.nih.gov/pubmed/?term=Shimizu%20N%5BAuthor%5D&cauthor=true&cauthor_uid=25836008). Effects of tooth root contact on the stability of orthodontic anchor screws in the maxilla: Comparison between self-drilling and self-tapping methods. [Am J Orthod Dentofacial Orthop.](http://www.ncbi.nlm.nih.gov/pubmed/25836008) 2015;147(4):483-91.

**Janson 2013**

[Janson G](http://www.ncbi.nlm.nih.gov/pubmed/?term=Janson%20G%5BAuthor%5D&cauthor=true&cauthor_uid=23062958), [Gigliotti MP](http://www.ncbi.nlm.nih.gov/pubmed/?term=Gigliotti%20MP%5BAuthor%5D&cauthor=true&cauthor_uid=23062958), [Estelita S](http://www.ncbi.nlm.nih.gov/pubmed/?term=Estelita%20S%5BAuthor%5D&cauthor=true&cauthor_uid=23062958), [Chiqueto K](http://www.ncbi.nlm.nih.gov/pubmed/?term=Chiqueto%20K%5BAuthor%5D&cauthor=true&cauthor_uid=23062958). Influence of miniscrew dental root proximity on its degree of late stability. [Int J Oral Maxillofac Surg.](http://www.ncbi.nlm.nih.gov/pubmed/?term=influence+of+miniscrew+dental+root+proximity) 2013 Apr;42(4):527-34.

**Kang 2009**

Kang YG, Kim JY, Lee YJ, Chung KR, Park YG. Stability of mini-screws invading the dental roots and their impact on the paradental tissues in beagles. Angle Orthod 2009;79:248-55.

**Kau 2010**

Kau CH, English JD, Muller-Delgardo MG, Hamid H, Ellis RK, Winklemann S. [Retrospective cone-beam computed tomography evaluation of temporary anchorage devices.](http://www.ncbi.nlm.nih.gov/pubmed/20152667) Am J Orthod Dentofacial Orthop. 2010;137(2):166.e1-5.

**Kim 2010**

Kim SH, Kang SM, Choi YS, Kook YA, Chung KR, Huang JC. [Cone-beam computed tomography evaluation of mini-implants after placement: Is root proximity a major risk factor for failure?](http://www.ncbi.nlm.nih.gov/pubmed/20816295) Am J Orthod Dentofacial Orthop. 2010 Sep;138(3):264-76.

**Kim 2011**

Kim H, Kim TW. [Histologic evaluation of root-surface healing after root contact or approximation during placement of mini-implants.](http://www.ncbi.nlm.nih.gov/pubmed/21640881) Am J Orthod Dentofacial Orthop. 2011 Jun;139(6):752-60.

**McManus 2010**

Effect of mini-screw maximum insertion torque on skeletal anchorage. MSc thesis. Submitted to the Graduate college of the University of Iowa in partial fulfillment of the requirements for the degree of Master of Science degree in orthodontics. University of Iowa (USA), Department of Orthodontics; 2010.

**Motoyoshi 2006**

Motoyoshi M, Hirabayashi M, Uemura M, Shimizu N. Recommended placement torque when tightening an orthodontic mini-implant. Clin Oral Implants Res. 2006;17:109-14.

**Motoyoshi 2007a**

Motoyoshi M, Matsuoka M, Shimizu N. Application of orthodontic mini-implants in adolescents. Int J Oral Maxillofac Surg. 2007;36:695-9.

**Motoyoshi 2007b**

Motoyoshi M, Yoshida T, Ono A, Shimizu N. Effect of cortical bone thickness and implant placement torque on stability of orthodontic mini-implants. Int J Oral Maxillofac Implants. 2007;22:779-84.

**Motoyoshi 2010**

Motoyoshi M, Uemura M, Ono A, Okazaki K, Shigeeda T, Shimizu N. Factors affecting the long-term stability of orthodontic mini-implants. Am J Orthod Dentofacial Orthop. 2010;137:588.e1-5.

**Shigeeda 2014**

[Shigeeda T](http://www.ncbi.nlm.nih.gov/pubmed/?term=Shigeeda%20T%5BAuthor%5D&cauthor=true&cauthor_uid=24739709). Root proximity and stability of orthodontic anchor screws. [J Oral Sci.](http://www.ncbi.nlm.nih.gov/pubmed/?term=Root+proximity+and+stability+of+orthodontic+anchor+screws) 2014;56(1):59-65.

**Shinohara 2013**

[Shinohara A](http://www.ncbi.nlm.nih.gov/pubmed/?term=Shinohara%20A%5BAuthor%5D&cauthor=true&cauthor_uid=23810045), [Motoyoshi M](http://www.ncbi.nlm.nih.gov/pubmed/?term=Motoyoshi%20M%5BAuthor%5D&cauthor=true&cauthor_uid=23810045), [Uchida Y](http://www.ncbi.nlm.nih.gov/pubmed/?term=Uchida%20Y%5BAuthor%5D&cauthor=true&cauthor_uid=23810045), [Shimizu N](http://www.ncbi.nlm.nih.gov/pubmed/?term=Shimizu%20N%5BAuthor%5D&cauthor=true&cauthor_uid=23810045). Root proximity and inclination of orthodontic mini-implants after placement: cone-beam computed tomography evaluation. [Am J Orthod Dentofacial Orthop.](http://www.ncbi.nlm.nih.gov/pubmed/?term=2)%09Root+proximity+and+inclination+of+orthodontic+mini-implants+after+placement%3A+Cone-beam+computed+tomography+evaluation) 2013 Jul;144(1):50-6.

**Son 2014**

Son S, Motoyoshi M, Uchida Y, Shimizu N. [Comparative study of the primary stability of self-drilling and self-tapping orthodontic miniscrews.](http://www.ncbi.nlm.nih.gov/pubmed/24703286) Am J Orthod Dentofacial Orthop. 2014 Apr;145(4):480-5.

**Suzuki 2011**

Suzuki EY, Suzuki B. Placement and removal torque values of orthodontic miniscrew implants. Am J Orthod Dentofacial Orthop. 2011;139:669-78.

**Suzuki 2013**

Suzuki M, Deguchi T, Watanabe H, Seiryu M, Iikubo M, Sasano T, Fujiyama K, Takano-Yamamoto T.

[Evaluation of optimal length and insertion torque for miniscrews.](http://www.ncbi.nlm.nih.gov/pubmed/23910206) Am J Orthod Dentofacial Orthop. 2013 Aug;144(2):251-9.

**Watanabe 2013**

Watanabe H, Deguchi T, Hasegawa M, Ito M, Kim S, Takano-Yamamoto T. [Orthodontic miniscrew failure rate and root proximity, insertion angle, bone contact length, and bone density.](http://www.ncbi.nlm.nih.gov/pubmed/23311659) Orthod Craniofac Res. 2013 Feb;16(1):44-55.

**Wilmes 2008**

[Wilmes B](http://www.ncbi.nlm.nih.gov/pubmed/?term=Wilmes%20B%5BAuthor%5D&cauthor=true&cauthor_uid=18947280), [Su YY](http://www.ncbi.nlm.nih.gov/pubmed/?term=Su%20YY%5BAuthor%5D&cauthor=true&cauthor_uid=18947280), [Drescher D](http://www.ncbi.nlm.nih.gov/pubmed/?term=Drescher%20D%5BAuthor%5D&cauthor=true&cauthor_uid=18947280). Insertion angle impact on primary stability of orthodontic mini-implants. [Angle Orthod.](http://www.ncbi.nlm.nih.gov/pubmed/18947280) 2008 Nov;78(6):1065-70.

**Wilmes 2011**

Wilmes B, Drescher D. [Impact of bone quality, implant type, and implantation site preparation on insertion torques of mini-implants used for orthodontic anchorage.](http://www.ncbi.nlm.nih.gov/pubmed/21458232)

Int J Oral Maxillofac Surg. 2011 Jul;40(7):697-703.
