# Supplementary material for: Insertion torque recordings for the diagnosis of contact between orthodontic mini-implants and dental roots: a systematic review
Source: Syst Rev. 2016 Mar 31;5:50. doi: 10.1186/s13643-016-0227-3 (PMC4818448; doi:10.1186/s13643-016-0227-3)
Supplement: Additional file 5: — Rationales for the ACROBAT-NRSI risk of bias scores. (DOCX 29 kb) [file 13643_2016_227_MOESM5_ESM.docx]

**Additional file 5. Rationales for the ACROBAT-NRSI risk of bias scores**

**Preliminary consideration of confounders**

**Patient related confounders**

Factors that relate to e.g. pre-existing disease (general health status), dental status, presence of comorbidities, health care utilization, adiposity, age, sex, and socioeconomic status, switches between interventions of interest, mobility, disabilities, ethnicity.

**Implant related confounders**

Factors that relate to e.g. the implant type, form, material, diameter, length, surface finish, drilling design, thread characteristics, pitch dimensions, flute in tip, flute in core, and being sterilized by an implant company or not.

**Location related confounders**

Factors that relate to e.g. the implant site , distance to root, assessment of bone condition, bone thickness, keratinized/non keratinized mucosa, mucosal thickness, and exposed/unexposed implants.

**Surgery related confounders**

Factors that relate to e.g. the experience of the operator, one or multiple operators, flap or flapless surgery, self-drilling or predrilling insertion technique, dimensions of the pilot holes for the self-drilling technique, predrilling device for the self-drilling pilot holes, predrilling speed for the self-drilling pilot holes, dimensions of the pilot holes for the pre-drilling technique, predrilling device for the predrilling pilot holes, predrilling speed for the pre-drilling pilot holes, distance between the screws, insertion depth, friction between the head of the screw and the bone, the direction of insertion, insertion speed, axial insertion force, insertion with insertion torqueing device, type of torqueing device, stripping of the bone, and mono or bicortical anchorage.

**Orthodontic related confounders**

Factors that relate to e.g. the type of orthodontic movement, time of force application, force magnitude, type of force, duration of force application, and the direction of force. Outcomes can change as a result of orthodontic treatment prior to implant insertion and recording of torque values for the index test. Outcomes could also change when orthodontic treatment is started after the recording of the index test, but prior to conducting the reference standard.

**Preliminary consideration of co-interventions**

The application of orthodontic forces prior to conducting the reference standard. Other types of co-interventions are highly unlikely, because the intervention and the outcome assessment are conducted within a short time span (generally not longer than 1 hour).

**Table 1. ACROBAT-NRSI risk of bias assessment and rationale for the study by Motoyoshi 2014 *[6]**

| **Domain** | **Risk of bias assessment *** | **Rationale for the risk of bias assessment** |
| --- | --- | --- |
| Bias due to confounding | Serious risk | Confounding could be a result of several items listed under ‘Preliminary consideration of confounders’.  Confounding was not controlled by the study design, because this study was not a randomized controlled trial.  Confounding was also not controlled through pre-specified eligibility criteria or statistical analyses. |
| Bias in selection of participants into the study | No information | No information was reported about the selection of patients into the research study. For example, it was not reported whether the patients were consecutively treated. This study probably had a retrospective design. |
| Bias in measurements of interventions | Moderate risk | The intervention status is well defined and it is highly unlikely that the intervention was misclassified. For example: the digital insertion torque measurements (index test) and the 3D radiographs (reference standard) were likely to correctly classify the target condition. However, the reference standard was recorded after loading of the OMIs, which could have influenced outcomes. |
| Bias due to departures from intended interventions | Low risk | There we no departures from intended interventions. |
| Bias due to missing data | Low risk | Everyone followed up through records. |
| Bias in measurement of outcomes | Moderate risk | Areas of concern for this item refer to:   - One examiner assessed implant-root contact. - Calibration and blinding of the outcome assessor was not reported. |
| Bias in selection of the reported result | Low risk | No areas of concern were identified for this item. |
| Overall | Serious risk | At least one domain was scored as ‘serious’ risk of bias, which means that the study as a whole has a risk of bias at least this severe. |

****Low risk of bias; Moderate risk of bias; Serious risk of bias; Critical risk of bias; No information on which to base a judgment about risk of bias***

**Table 2. ACROBAT-NRSI risk of bias assessment and rationale for the study by Chen 2008 *[84]**

| **Domain** | **Risk of bias assessment *** | **Rationale for the risk of bias assessment** |
| --- | --- | --- |
| Bias due to confounding | Moderate risk | Confounding could be a result of several items listed under ‘Preliminary consideration of confounders’. It was not clear whether confounding was controlled by the study design, because randomization procedures were not transparent. ‘Randomly chosen’ does not mean ‘randomization’. Confounding was also not controlled through pre-specified eligibility criteria or statistical analyses. |
| Bias in selection of participants into the study | Low risk | No areas of concern were identified for this item. |
| Bias in measurements of interventions | Serious risk | Areas of concern for this item refer to:   - Pre-drilling procedures deflate sensitivity. - Different insertion sites for implants with and without root contact. - Hitting a root on purpose could give different torque values than those obtained when just glancing a root. - The use of a mechanical torque driver. - Maximum insertion torque was only measured during the last 1/3 of the insertion path. - The reference standard was not measured at standardized time points. - Unclear definition of the target condition. |
| Bias due to departures from intended interventions | Serious risk | Departure from the use of radiography to histology for the diagnosis of the target condition as a result of the inaccuracy of the 2D radiographs of 32 of 72 OMIs. |
| Bias due to missing data | Low risk | Everyone followed up through records. |
| Bias in measurement of outcomes | No information | Areas of concern for this item refer to the lack of reporting on blinding and calibration of outcome assessors. |
| Bias in selection of the reported result | Low risk | No areas of concern were identified for this item. |
| Overall | Serious risk | At least one domain was scored as ‘serious’ risk of bias, which means that the study as a whole has a risk of bias at least as severe. |

****Low risk of bias; Moderate risk of bias; Serious risk of bias; Critical risk of bias; No information on which to base a judgment about risk of bias***

**Table 3. ACROBAT-NRSI risk of bias assessment and rationale for the study by Brisceno 2009 *[25]**

| **Domain** | **Risk of bias assessment *** | **Rationale for the risk of bias assessment** |
| --- | --- | --- |
| Bias due to confounding | Serious risk | Confounding could be a result of several items listed under ‘Preliminary consideration of confounders’.  Confounding was not controlled by the study design, because, left and right sides of the mandible were randomized for 6 or 12 weeks of healing after implant insertion. These sites were not randomized for our specific research questions. Confounding was also not controlled through pre-specified eligibility criteria or statistical analyses. |
| Bias in selection of participants into the study | Low risk | No areas of concern were identified for this item. |
| Bias in measurements of interventions | Serious risk | Areas of concern for this item refer to:   - The time point of the maximum insertion torque recording was not reported. - Surgery related variables were underreported; for example: implants were inserted interradicularly at many different sites, but these procedures were not standardized. - Hitting a root on purpose could give different torque values than those obtained when just glancing a root. - OMIs were inserted at different time points in a split mouth model. - The inaccuracy of 2D radiographs. |
| Bias due to departures from intended interventions | Low risk | There we no departures from intended interventions. |
| Bias due to missing data | Serious risk | It was impossible to draw a flow diagram of the participants |
| Bias in measurement of outcomes | No information | Areas of concern for this item refer to the lack of reporting on blinding and calibration of outcome assessors. |
| Bias in selection of the reported result | Low risk | No areas of concern were identified for this item. |
| Overall | Serious risk | At least one domain was scored as ‘serious’ risk of bias, which means that the study as a whole has a risk of bias at least as severe. |

****Low risk of bias; Moderate risk of bias; Serious risk of bias; Critical risk of bias; No information on which to base a judgment about risk of bias***

**Table 4. ACROBAT-NRSI risk of bias assessment and rationale for the study by Wilmes 2008 *[33]**

| **Domain** | **Risk of bias assessment *** | **Rationale for the risk of bias assessment** |
| --- | --- | --- |
| Bias due to confounding | Moderate risk | Confounding could be a result of several items listed under ‘Preliminary consideration of confounders’.  Confounding was not controlled by the study design, because randomization procedures were not mentioned.  Confounding was also not controlled through pre-specified eligibility criteria or statistical analyses. |
| Bias in selection of participants into the study | Low risk | No areas of concern were identified for this item. |
| Bias in measurements of interventions | Serious risk | Areas of concern for this item refer to:   - Pre-drilling procedures deflate sensitivity. - Small (4 mm) inter-implant distances. - Several surgery related factors were not reported. - Hitting a root on purpose could give different torque values than those obtained when just glancing a root. |
| Bias due to departures from intended interventions | Low risk | There we no departures from intended interventions. |
| Bias due to missing data | Serious risk | The loss of 15 of 320 implants was not explained. |
| Bias in measurement of outcomes | No information | Areas of concern for this item refer to the lack of reporting on blinding and calibration of outcome assessors. |
| Bias in selection of the reported result | Low risk | No areas of concern were identified for this item. |
| Overall | Serious risk | At least one domain was scored as ‘serious’ risk of bias, which means that the study as a whole has a risk of bias at least as severe. |

****Low risk of bias; Moderate risk of bias; Serious risk of bias; Critical risk of bias; No information on which to base a judgment about risk of bias***

**Table 5. ACROBAT-NRSI risk of bias assessment and rationale for the study by McEwan 2012 *[32]**

| **Domain** | **Risk of bias assessment *** | **Rationale for the risk of bias assessment** |
| --- | --- | --- |
| Bias due to confounding | Moderate risk | Confounding could be a result of several items listed under ‘Preliminary consideration of confounders’. Confounding was not controlled by the study design, because randomization procedures were not mentioned. Confounding was also not controlled through pre-specified eligibility criteria or statistical analyses. |
| Bias in selection of participants into the study | Low risk | No areas of concern were identified for this item. |
| Bias in measurements of interventions | Moderate risk | Areas of concern for this item refer to:   - Torque was measured during a standardized time period, but was not standardized for the insertion depth - Some surgery related factors were not reported |
| Bias due to departures from intended interventions | Low risk | There we no departures from intended interventions. |
| Bias due to missing data | Moderate risk | In one implant group 3 of the 10 implants fractured and were therefore excluded from analysis. This exclusion was appropriate, but its consequences could be problematic in the context of the small sample size. |
| Bias in measurement of outcomes | No information | Areas of concern for this item refer to the lack of reporting on blinding and calibration of outcome assessors. |
| Bias in selection of the reported result | Low risk | No areas of concern were identified for this item. |
| Overall | Moderate risk | At least one domain was scored as ‘moderate’ risk of bias, which means that the study as a whole has a risk of bias at least as severe |

****Low risk of bias; Moderate risk of bias; Serious risk of bias; Critical risk of bias; No information on which to base a judgment about risk of bias***
