# Supplementary material for: Insertion torque recordings for the diagnosis of contact between orthodontic mini-implants and dental roots: a systematic review
Source: Syst Rev. 2016 Mar 31;5:50. doi: 10.1186/s13643-016-0227-3 (PMC4818448; doi:10.1186/s13643-016-0227-3)
Supplement: Additional file 6: — Exemplary emails for contacting authors. (DOCX 18 kb) [file 13643_2016_227_MOESM6_ESM.docx]

**Additional file 6. Exemplary emails for contacting authors**

**Exemplary email to assess author’s willingness to reply to questions of systematic reviewers**

| Dear Professor ………….,  We are currently conducting a systematic review* on orthodontic mini implants and would like to obtain some additional information on your article:    “ …………………………………………………………….”  In the near future we would like to ask you some simple questions on this publication. Answering these questions will probably not require more than 2 minutes of your time.  In this email we are only interested to find out whether you are willing to provide us with this additional information or not.  We therefore ask you the following question:  **Are you in the near future willing to respond to some simple questions pertinent to the indicated article ?**  - If you are willing to respond to our questions please reply to this email, a "YES" is enough.    - If you are not willing to respond to our questions please respond this email with a “NO”. In case, you do not reply, we will send you a reminder email 14 days after the current email.  -In case you respond with a “NO” or do not reply to this email and to the reminder, we will not further contact you, but will contact at least one of the other co-authors of your research study.  Thank you very much for your cooperation.  Sincerely,  Reint Meursinge Reynders and Nicola Di Girolamo  ----------------------------------------------------- *This systematic review originated as an assignment for the Evidence Based Health Care program at the University of Oxford, UK. The aforementioned individuals are subjectively responsible for the information requested. |
| --- |

**Exemplary email to authors to obtain additional data of their research studies**

| Dear Professor ………..,  In an earlier mail we asked you to answer some simple questions on your research article listed under here.  Your answers could be a great contribution to improve on the quality of our systematic review*. The published protocol of our systematic review can be found in the attachment.  As we wrote in our previous email we would like to ask you some (6 in total) short questions regarding your article:  “ …………………………………………………………….”  All questions are combined closed-and open-ended questions.  If you answer a question with a YES, we would like you to give a short description.  Definitions of specific domains are presented under the Question and Answer table.   \| **Questions** \| **Answer** \| \| --- \| --- \| \| ***Was a random sequence generated^1^ to allocate participants ?***  If you answer with a **YES**, could you please describe how this procedure was conducted  If you answer with a **NO**, it is not necessary to provide any additional information \|  \| \| ***Was allocation concealed^2^ ?***  If you answer with a **YES**, could you please describe how this procedure was conducted  If you answer with a **NO**, it is not necessary to provide any additional information \|  \| \| ***Were participants and personnel blinded^3^ ?***  If you answer with a **YES**, could you please describe how this procedure was conducted  If you answer with a **NO**, it is not necessary to provide any additional information \|  \| \| ***Were outcome assessors blinded^4^ ?***  If you answer with a **YES**, could you please describe how this procedure was conducted  If you answer with a **NO**, it is not necessary to provide any additional information \|  \| \| ***Were participants consecutively treated^5^ ?***  If you answer with a **YES**, could you please describe how this procedure was conducted  If you answer with a **NO**, it is not necessary to provide any additional information \|  \| \| ***Were the implants loaded with orthodontic forces prior to taking the radiographic images ?***  If you answer this question with a **YES**, could you please describe how this procedure was conducted.  If you answer this question with a **NO**, it is not necessary to provide any additional information \|  \|   **Definitions of the various domains:**  **^1^Random sequence generation:**  This domain refers to the generation of a random sequence for allocating participants to different treatment groups.   Tools for random sequence generation refer to: 1) random number tables; 2) computer random number generation; 3) coin tossing; 4) shuffling cards or envelopes; 5) throwing dice etc. (Higgins 2011).  **^2^Allocation concealment**  This domain refers to methods to conceal that participants and investigators enrolling participants can foresee the allocation to a particular treatment group.  Tools for allocation concealment refer to: 1) central allocation (e.g. including telephone, internet-based and pharmacy-controlled randomization); 2) sequentially numbered drug containers with an identical appearance; 3) sequentially numbered, non transparent, and sealed envelopes (Higgins 2011)**. Allocation concealment covers the period from the moment of the random sequence generation until the start of conducting the interventional procedures.  **^3^Blinding of participants and personnel**  This domain refers to blinding of participants and personnel to avoid knowledge of the allocation to a specific study arm.  This domain covers all blinding issues from the moment of allocation to a specific treatment arm until the completion of the final research procedures, but does not cover blinding issues during the assessment of outcomes.  **^4^Blinding  of outcome assessors**  This domain refers to blinding of outcome assessors to avoid knowledge of the allocation to a specific study arm.  This domain covers exclusively blinding issues during the periods of recording outcomes.  **^5^Consecutively treated participants**  This domain refers to the inclusion of all participants treated over a period of time.  Non-consecutively treated participants refers to a selection of participants.  We thank you again for your help and time.  We will send you a copy of our systematic review as soon as it will be accepted for publication.  In the case that you have changed your mind and are not willing to reply to these questions, could you please explain the rationale for this decision.  Thank you again very much for your cooperation.  Sincerely,  Reint Meursinge Reynders and Nicola Di Girolamo  -------------------------------------------------------------------------------------------------------------------------------- *This systematic review originated as an assignment for the Evidence Based Health Care program at the University of Oxford, UK.  The aforementioned individuals are subjectively responsible for the information requested.  **Higgins 2011  Higgins JPT, Altman DG, Sterne JAC (editors). Chapter 8: Assessing risk of bias in included studies. In: Higgins JPT, Green S (editors). Cochrane Handbook for Systematic Reviews of Interventions Version 5.1.0 (updated March 2011). The Cochrane Collaboration, 2011. [online]  Available from:  [www.cochrane-handbook.org](http://www.cochrane-handbook.org). (accessed October 10^th^ 2014). |
| --- | --- | --- | --- | --- | --- | --- | --- | --- | --- | --- | --- | --- | --- | --- |
