# Supplementary material for: Insertion torque recordings for the diagnosis of contact between orthodontic mini-implants and dental roots: a systematic review
Source: Syst Rev. 2016 Mar 31;5:50. doi: 10.1186/s13643-016-0227-3 (PMC4818448; doi:10.1186/s13643-016-0227-3)
Supplement: Additional file 7: — Outcomes of contacting authors. (DOCX 16 kb) [file 13643_2016_227_MOESM7_ESM.docx]

**Additional file 7. Outcomes of contacting authors**

**Table 1. Outcomes of author’s willingness to reply to questions of systematic reviewers**

| **Article** | **Contacted author(s)** | **Number of contacting attempts and time to get a response*** | **Reminder mails?** | **Co-authors contacted?** | **Willingness to reply ?** |
| --- | --- | --- | --- | --- | --- |
| Motoyoshi 2014 [6] | Motoyoshi M. | 1 attempt  Response in 1 day | No | No | Unclear** |
| Chen 2008  [84] | Yao CC. and Chen YJ. | 2 attempts  Response after 25 days | Yes | Yes | Yes |
| Brisceno 2009 [25] | Buschang PH. and  Rossouw PE. | 3 attempts  Response after 25 days | Yes | Yes | Yes |
| Wilmes 2008 [33] | Wilmes B. | 1 attempt  Response after 1 day | No | No | Yes |
| McEwan 2012 [32] | McEwan MB. | 1 attempt  Response the same day | No | No | Yes |

* This number refers to the total number of attempts by email to get an answer from a contacted author.

This number also includes the number of attempts to contact a co-author(s). An initial attempt or a

subsequent reminder attempt are each counted as 1 attempt. As soon as authors have replied, successive

emails are not counted as additional attempts. Ideally only 1 attempts is made.

Attempts of sending emails from other email addresses are not counted as additional attempts. Sending

such emails could at times be indicated, because our initial email could be identified as “spam mail” and

could then be deleted by the receiving internet provider.

** Dr. Motoyoshi explained that his willingness to reply to questions “will depend on the questions asked”.

**Table 2. Outcomes of answering by contacted authors to research questions of systematic reviewers**

| **Article** | **Contacted author(s)** | **Number of contacting attempts to get a reply to research questions*** | **Reminder mails?** | **Co-authors contacted?** | **Number of research questions answered** |
| --- | --- | --- | --- | --- | --- |
| Motoyoshi 2014 [6] | Motoyoshi M. and Inaba M | 4 attempts | Yes | Yes | 0 of 6 questions** |
| Chen 2008 [84] | Yao CC. | 1 attempt | No | No | 1 of 1 question |
| Brisceno 2009 [25] | Buschang PH. | 4 attempts | Yes | No | 6 of 6 questions |
| Wilmes 2008 [33] | Wilmes B. and Sadigh L | 4 attempts | Yes | Yes | 2 of 6 questions*** |
| McEwan 2012 [32] | McEwan MB. | 1 attempt | No | No | 7 of 7 questions |

* This number refers to the total number of attempts by email to get an answer from a contacted author.

This number also includes the number of attempts to contact a co-author(s). An initial attempt or a

subsequent reminder attempt are each counted as 1 attempt. As soon as authors have replied, successive

emails are not counted as additional attempts. Ideally only 1 attempts is made.

Attempts of sending emails from other email addresses are not counted as additional attempts. Sending

such emails could at times be indicated, because our initial email could be identified as “spam mail” and

could then be deleted by the receiving internet provider.

** Dr. Motoyoshi did not respond to our research questions, because he felt that his study was not eligible

for our systematic review.

***Dr. Wilmes addressed 2 out of 6 questions. He explained that we could obtain additional answers from

a co-author, Dr. Sadigh, and he provided us with her email address. We contacted Dr. Sadigh, but the

email address was incorrect. We subsequently contacted Dr. Wilmes about this issue and he provided us

with a new email address. We subsequently contacted Dr. Sadigh and also Dr. Wilmes, but both authors

did not reply.
